# Transduction of the Geomagnetic Field as Evidenced from alpha-Band Activity in the Human Brain

**DOI:** 10.1523/ENEURO.0483-18.2019

**Published:** 2019-04-26

**Authors:** Connie X. Wang, Isaac A. Hilburn, Daw-An Wu, Yuki Mizuhara, Christopher P. Cousté, Jacob N. H. Abrahams, Sam E. Bernstein, Ayumu Matani, Shinsuke Shimojo, Joseph L. Kirschvink

**Affiliations:** 1 Computation and Neural Systems, California Institute of Technology, Pasadena, CA, 91125; 2 Division of Geological and Planetary Sciences, California Institute of Technology, Pasadena, CA, 91125; 3 Division of Biology and Biological Engineering, California Institute of Technology, Pasadena, CA, 91125; 4 Graduate School of Information Science and Technology, The University of Tokyo, Bunkyo-ku, Tokyo 113-8654, Japan; 5 Department of Computer Science, Princeton University, Princeton, NJ 08544; 6 Tohoku University Graduate School of Life Sciences, Sendai, Miyagi 980-8577, Japan; 7 Earth-Life Science Institute, Tokyo Institute of Technology, Meguro, Tokyo 152-8550, Japan

**Keywords:** alpha-ERD, biogenic magnetite, biophysics, EEG, magnetoreception, quantum compass

## Abstract

Magnetoreception, the perception of the geomagnetic field, is a sensory modality well-established across all major groups of vertebrates and some invertebrates, but its presence in humans has been tested rarely, yielding inconclusive results. We report here a strong, specific human brain response to ecologically-relevant rotations of Earth-strength magnetic fields. Following geomagnetic stimulation, a drop in amplitude of electroencephalography (EEG) alpha-oscillations (8–13 Hz) occurred in a repeatable manner. Termed alpha-event-related desynchronization (alpha-ERD), such a response has been associated previously with sensory and cognitive processing of external stimuli including vision, auditory and somatosensory cues. Alpha-ERD in response to the geomagnetic field was triggered only by horizontal rotations when the static vertical magnetic field was directed downwards, as it is in the Northern Hemisphere; no brain responses were elicited by the same horizontal rotations when the static vertical component was directed upwards. This implicates a biological response tuned to the ecology of the local human population, rather than a generic physical effect. Biophysical tests showed that the neural response was sensitive to static components of the magnetic field. This rules out all forms of electrical induction (including artifacts from the electrodes) which are determined solely on dynamic components of the field. The neural response was also sensitive to the polarity of the magnetic field. This rules out free-radical “quantum compass” mechanisms like the cryptochrome hypothesis, which can detect only axial alignment. Ferromagnetism remains a viable biophysical mechanism for sensory transduction and provides a basis to start the behavioral exploration of human magnetoreception.

## Visual Abstract

**Figure:**
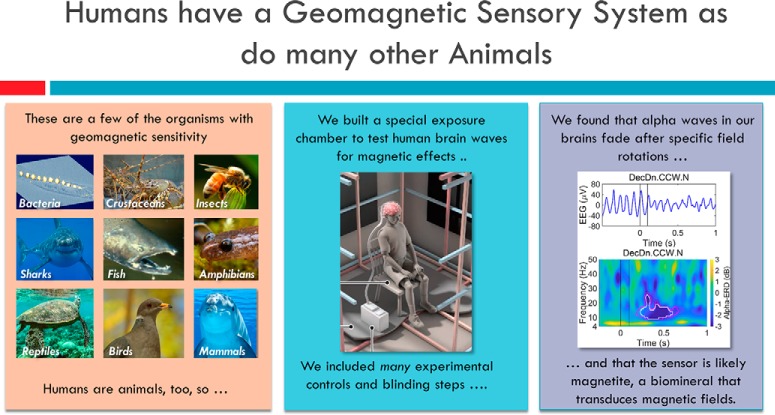

## Significance Statement

Although many migrating and homing animals are sensitive to Earth’s magnetic field, most humans are not consciously aware of the geomagnetic stimuli that we encounter in everyday life. Either we have lost a shared, ancestral magnetosensory system, or the system lacks a conscious component with detectable neural activity but no apparent perceptual awareness by us. We found two classes of ecologically-relevant rotations of Earth-strength magnetic fields that produce strong, specific and repeatable effects on human brainwave activity in the electroencephalography (EEG) alpha-band (8–13 Hz); EEG discriminates in response to different geomagnetic field stimuli. Biophysical tests rule out all except the presence of a ferromagnetic transduction element, such as biologically-precipitated crystals of magnetite (Fe_3_O_4_).

## Introduction

Magnetoreception is a well-known sensory modality in bacteria (Frankel and Blakemore, 1980), protozoans (Bazylinski et al., 2000) and a variety of animals (Wiltschko and Wiltschko, 1995a; Walker et al., 2002; Johnsen and Lohmann, 2008), but whether humans have this ancient sensory system has never been conclusively established. Behavioral results suggesting that geomagnetic fields influence human orientation during displacement experiments (Baker, 1980, 1982, 1987) were not replicated (Gould and Able, 1981; Able and Gergits, 1985; Westby and Partridge, 1986). Attempts to detect human brain responses using electroencephalography (EEG) were limited by the computational methods that were used (Sastre et al., 2002). Twenty to 30 years after these previous flurries of research, the question of human magnetoreception remains unanswered.

In the meantime, there have been major advances in our understanding of animal geomagnetic sensory systems. An ever-expanding list of experiments on magnetically-sensitive organisms has revealed physiologically-relevant stimuli as well as environmental factors that may interfere with magnetosensory processing (Wiltschko and Wiltschko, 1995a; Lohmann et al., 2001; Walker et al., 2002). Animal findings provide a potential feature space for exploring human magnetoreception, the physical parameters and coordinate frames to be manipulated in human testing (Wiltschko, 1972; Kirschvink et al., 1997). In animals, geomagnetic navigation is thought to involve both a compass and map response (Kramer, 1953). The compass response simply uses the geomagnetic field as an indicator to orient the animal relative to the local magnetic north/south direction (Wiltschko and Wiltschko, 1995a; Lohmann et al., 2001). The magnetic map is a more complex response involving various components of field intensity and direction; direction is further subdivided into inclination (vertical angle from the horizontal plane; the North-seeking vector of the geomagnetic field dips downwards in the Northern Hemisphere) and declination (clockwise angle of the horizontal component from Geographic North, as in a man-made compass). Notably, magnetosensory responses tend to shut down altogether in the presence of anomalies (e.g., sunspot activity or local geomagnetic irregularities) that cause the local magnetic field to deviate significantly from typical ambient values (Wiltschko, 1972; Martin and Lindauer, 1977), an adaptation that is thought to guard against navigational errors. These results indicate that geomagnetic cues are subject to complex neural processing, as in most other sensory systems.

Physiologic studies have flagged the ophthalmic branch of the trigeminal system (and equivalents) in fish (Walker et al., 1997), birds (Semm and Beason, 1990; Beason and Semm, 1996; Mora et al., 2004; Elbers et al., 2017), and rodents (Wegner et al., 2006) as a conduit of magnetic sensory information to the brain. In humans, the trigeminal system includes many autonomic, visceral, and proprioceptive functions that lie outside conscious awareness (Saper, 2002; Fillmore and Seifert, 2015). For example, the ophthalmic branch contains parasympathetic nerve fibers and carries signals of extraocular proprioception, which do not reach conscious awareness (Liu, 2005).

If the physiologic components of a magnetosensory system have been passed from animals to humans, then their function may be either subconscious or only weakly available to conscious perception. Behavioral experiments could be easily confounded by cognitive factors such as attention, memory and volition, making the results weak or difficult to replicate at the group or individual levels. Since brain activity underlies all behavior, we chose a more direct electrophysiological approach to test for the transduction of geomagnetic fields in humans.

## Materials and Methods

### Part 1: summary and design logic

#### Experimental equipment setup

**Figure 1.**
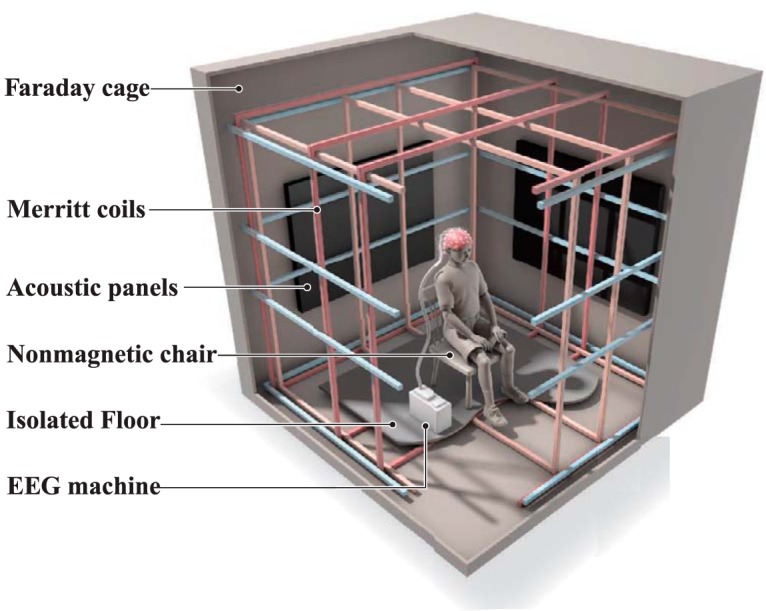
Schematic illustration of the experimental setup. The ∼1-mm-thick aluminum panels of the electrically-grounded Faraday shielding provides an electromagnetically “quiet” environment. Three orthogonal sets of square coils ∼2 m on edge, following the design of Merritt et al. (1983), allow the ambient geomagnetic field to be altered around the participant’s head with high spatial uniformity; double-wrapping provides an active-sham for blinding of experimental conditions (Kirschvink, 1992b). Acoustic panels on the wall help reduce external noise from the building air ventilation system as well as internal noise due to echoing. A non-magnetic chair is supported on an elevated wooden base isolated from direct contact with the magnetic coils. The battery-powered EEG is located on a stool behind the participant and communicates with the recording computer via an optical fiber cable to a control room ∼20 m away. Additional details are available in Figure 2. This diagram and the center figure for the visual abstract was modified from the figure “Center of attraction,” by C. Bickel (Hand, 2016), with permission.

**Figure 2.**
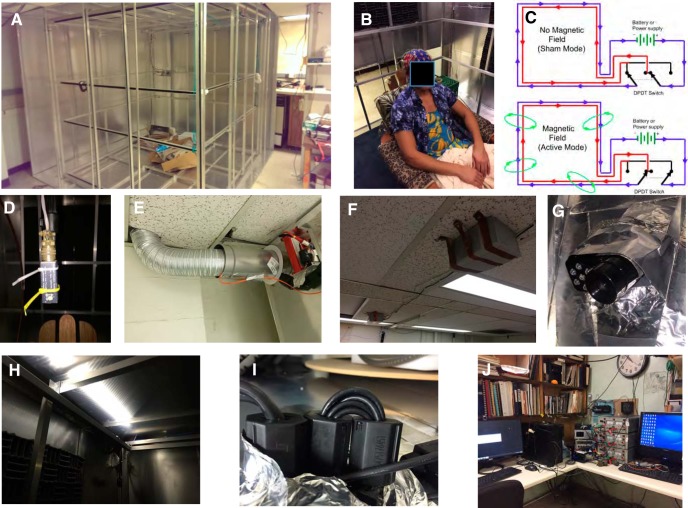
Additional images of critical aspects of the human magnetic exposure at Caltech. ***A***, Partially complete assembly of the Faraday cage (summer of 2014) showing the nested set of orthogonal, Merritt square four-coils (Merritt et al., 1983) with all but two aluminum walls of the Faraday cage complete. ***B***, Image of a participant in the facility seated in a comfortable, non-magnetic wooden chair and wearing the 64-lead BioSim EEG head cap. The EEG sensor leads are carefully braided together to minimize electrical artifacts. The chair is on a raised wooden platform that is isolated mechanically from the magnet coils and covered with a layer of synthetic carpeting; the height is such that the participant’s head is in the central area of highest magnetic field uniformity. ***C***, Schematic of the double-wrapped control circuits that allow active-sham experiments (Kirschvink, 1992b). In each axis of the coils, the four square frames are wrapped in series with two discrete strands of insulated copper magnet wire and with the number of turns and coil spacing chosen to produce a high-volume, uniform applied magnetic field (Merritt et al., 1983). Reversing the current flow in one of the wire strands via a DPDT switch results in cancellation of the external field with virtually all other parameters being the same. This scheme is implemented on all three independently controlled coil axes (Up/Down, East/West, and North/South). ***D***, Fluxgate magnetometer (Applied Physics Systems 520A) three-axis magnetic field sensor attached to a collapsing carbon-fiber camera stand mount. At the start of each session, the fluxgate is lowered to the center of the chamber for an initial current/control calibration of the ambient geomagnetic field. It is then raised to a position ∼30 cm above the participant’s head during the following experimental trials, and the three-axis magnetic field readings are recorded continuously in the same fashion as the EEG voltage signals. ***E***, Air duct. A 15 cm in diameter aluminum air duct ∼2-m-long connects a variable-speed (100 W) electric fan to the upper SE corner of the experimental chamber; this is also the conduit used for the major electrical cables (power for the magnetic coils, sensor leads for the fluxgate, etc.). ***F***, ***G***, An intercom/video monitoring system was devised by mounting a computer-controlled loudspeaker (***F***) outside the Faraday shield on the ceiling North of the chamber coupled with (***G***) a USB-linked IR video camera/microphone system mounted just inside the shield. Note the conductive aluminum tape shielding around the camera to reduce Rf interference. During all experimental trials a small DPDT relay located in the control room disconnects the speaker from computer and directly shorts the speaker connections. A second microphone in the control room can be switched on to communicate with the participant in the experimental chamber, as needed. An experimenter monitors the audio and video of participants at all times, as per Caltech IRB safety requirements. ***H***, LED lights, 12 VDC array, arranged to illuminate from the top surface of the magnetic coils near the ceiling of the chamber. These are powered by rechargeable 11.1-V lithium battery packs (visible in ***E***) and controlled by an external switch. ***I***, Ferrite chokes. Whenever possible, these are mounted in a multiple-turn figure-eight fashion (Counselman, 2013) on all conductive wires and cables entering the shielded area and supplemented with grounded aluminum wool when needed. ***J***, Image of the remote-control area including (from left to right): the PC for controlling the coils, the DPDT switches for changing between active and sham modes, the fluxgate control unit, the three power amplifiers that control the current in the remote coil room, and the separate PC that records the EEG data. Participants seated in the experimental chamber do not report being able to hear sounds from the control room and vice versa. Additional guidance for the design of biomagnetic experiments is given by Kirschvink et al. (2010) and Schwarze et al. (2016).

We constructed an isolated, radio frequency-shielded chamber wrapped with three nested sets of orthogonal square coils, using the four-coil design of Merritt et al. (1983) for high central field uniformity (Fig. 1; further details in Fig. 2 and Materials and Methods, Part 2: details for replication and validation). Each coil contained two matched sets of windings to allow operation in active or sham mode. In active mode, currents in paired windings were parallel, leading to summation of generated magnetic fields. In sham mode, currents ran antiparallel, yielding no measurable external field, but with similar ohmic heating and magnetomechanical effects as in active mode (Kirschvink, 1992b). Active and sham modes were toggled by manual switches in the distant control room, leaving computer and amplifier settings unchanged. Coils were housed within an acoustically-attenuated, grounded Faraday cage with aluminum panels forming the walls, floor and ceiling. Participants sat upright in a wooden chair on a platform electrically isolated from the coil system with their heads positioned near the center of the uniform field region. The magnetic field inside the experimental chamber was monitored by a three-axis Applied Physics Systems 520A fluxgate magnetometer. EEG was continuously recorded from 64 electrodes using a BioSemi ActiveTwo system with electrode positions coded in the International 10-20 System (e.g., Fz, CPz, etc.). Inside the cage, the battery-powered digital conversion unit relayed data over a non-conductive, optical fiber cable to a remote-control room, ∼20 m away, where all power supplies, computers and monitoring equipment were located.

#### Experimental sequence

A ∼1-h EEG session consisted of multiple ∼7-min experimental runs. In each run of 100+ trials, magnetic field direction rotated repeatedly between two preset orientations with field intensity held nearly constant at the ambient lab value (∼35 µT). In SWEEP trials, the magnetic field started in one orientation then rotated smoothly over 100 ms to the other orientation. As a control condition, FIXED trials with no magnetic field rotation were interspersed among SWEEP trials according to pseudorandom sequences generated by software. Trials were separated in time by 2–3 s.

#### Participant blinding

During experiments, participants sat with their eyes closed in total darkness. Participants were blind to active versus sham modes, trial sequences, and trial onset timings. The experimental chamber was dark, quiet and isolated from the control room during runs. Auditory tones signaled only the beginning and end of experiment runs, and experimenters only communicated with participants once or twice per session between active runs to update the participant on the number of runs remaining. When time allowed, sham runs were matched to active runs using the same software settings. Active and sham runs were programmatically identical, differing only in the position of hardware switches that directed current to run parallel or antiparallel through paired loops. Sham runs served as an additional control for non-magnetic sensory confounds, such as sub-aural stimuli or mechanical oscillations from the coil system.

#### Magnetic rotation stimuli

**Figure 3.**
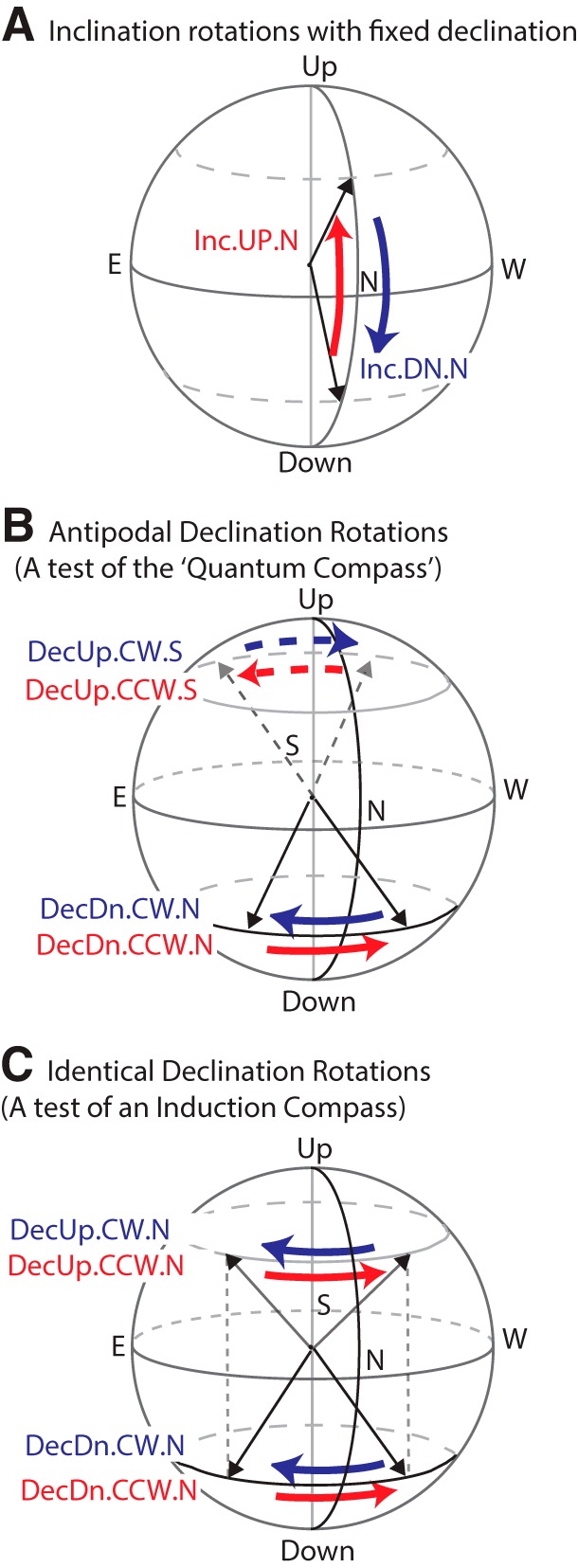
Magnetic field rotations used in these experiments. In the first ∼100 ms of each experimental trial, the magnetic field vector was either: (1) rotated from the first preset orientation to the second (SWEEP), (2) rotated from the second preset orientation to the first (also SWEEP), or (3) left unchanged (FIXED). In all experimental trials, the field intensity was held constant at the ambient lab value (∼35 μT). For declination rotations, the horizontal rotation angle was +90° or –90°. For inclination rotations, the vertical rotation angle was either +120°/–120°, or +150°/–150°, depending on the particular inclination rotation experiment. ***A***, Inclination rotations between ±60° and ±75°. The magnetic field vector rotates from downwards to upwards (Inc.UP.N, red) and vice versa (Inc.DN.N, blue), with declination steady at North (0°). ***B***, Declination rotations used in main assay (solid arrows) and vector opposite rotations used to test the quantum compass hypothesis (dashed arrows). In the main assay, the magnetic field rotated between NE (45°) and NW (315°) with inclination held downwards (+60° or +75°) as in the Northern Hemisphere (DecDn.CW.N and DecDn.CCW.N); vector opposites with upwards inclination (−60° or −75°) and declination rotations between SE (135°) and SW (225°) are shown with dashed arrows (DecUp.CW.S and DecUp.CCW.S). ***C***, Identical declination rotations, with static but opposite vertical components, used to test the electrical induction hypothesis. The magnetic field was shifted in the Northerly direction between NE (45°) and NW (315°) with inclination held downwards (+75°, DecDn.CW.N and DecDn.CCW.N) or upwards (−75°, DecUp.CW.S and DecUp.CCW.S). The two dotted vertical lines indicate that the rotations started at the same declination values. In both ***B***, ***C***, counterclockwise rotations (viewed from above) are shown in red, clockwise in blue.

Figure 3 shows the magnetic field rotations used. Note that experimental variables differing between runs are denoted in camel case as in DecDn, DecUp, active, sham, etc., whereas variables that change within runs are designated in all capitals like FIXED, SWEEP, CCW, CW, UP, DN, etc. In inclination (Inc) experiments (Fig. 3*A*), declination direction was fixed to North (0° declination in our coordinate system), and participants sat facing North. Rotation of the field vector from downwards to upwards was designated as an Inc.UP.N trial and the return sweep as Inc.DN.N, with UP/DN indicating the direction of field rotation. In declination (Dec) experiments (Fig. 3*B*,*C*), we held inclination (and hence the vertical component of the field vector) constant, while rotating the horizontal component clockwise or counterclockwise to vary the declination. For trials with downwards inclination (as in the Northern Hemisphere), field rotations swept the horizontal component 90° CW or CCW between Northeast and Northwest, designated as DecDn.CW.N or DecDn.CCW.N, respectively, with .N indicating a Northerly direction. To test biophysical hypotheses of magnetoreception as discussed below, we conducted additional declination rotation experiments with static, upwards inclination. As shown in Figure 3*B*, rotating an upwards-directed field vector between SE and SW (DecUp.CW.S and DecUp.CCW.S) antiparallel to the downwards-directed rotations provides tests of the quantum compass biophysical model, while sweeping an upwards vector between NE and NW (DecUp.CW.N and DecUp.CCW.N) provides a general test for electrical induction (Fig. 3*C*).

#### EEG artifact

**Figure 4.**
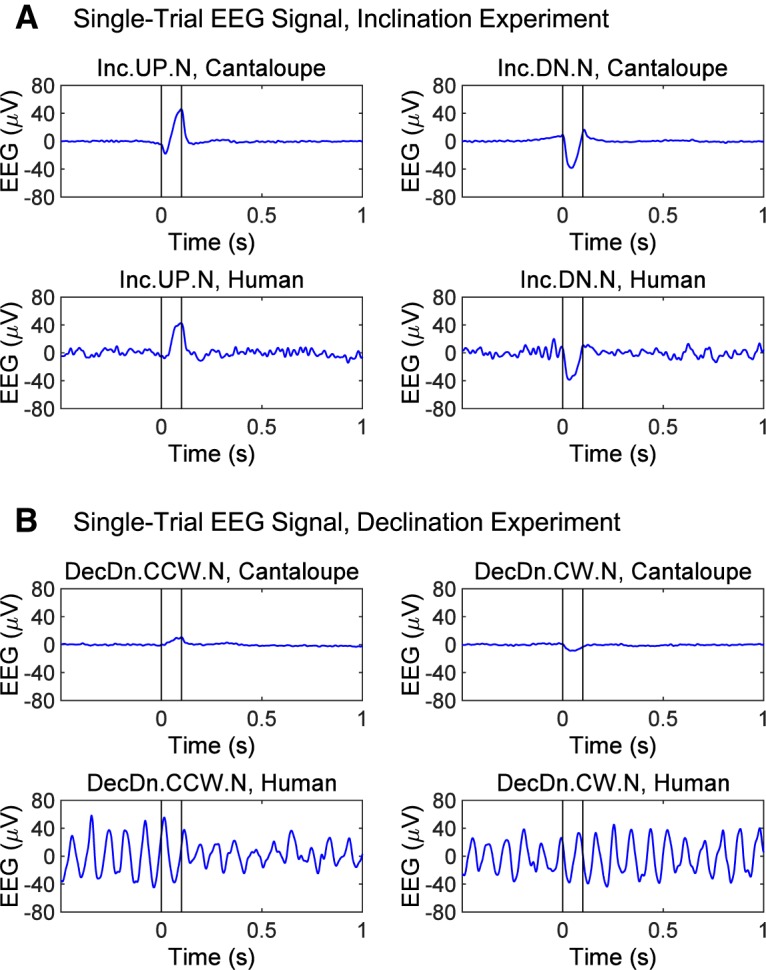
Examples of single-trial, time-domain, bandpass-filtered (1–50 Hz) EEG traces at electrode Fz from phantom (cantaloupe) and human participants (one with low and one with high baseline alpha-power) that illustrate the type of data gathered in this study. ***A***, Effect of a 0.1 s inclination sweep of a Northward-pointing, 35-µT magnetic field rotating between a dip of 75° down to 75° up (Inc.UP.N, left panels) and the reverse (Inc.DN.N, right panels). This is the largest stimulus used in our experiments (150° arc, effective frequency 4.2 Hz, with the full vector of 35-µT undergoing rotation). The cantaloupe records an ∼40-µV artifact during the sweep interval but is otherwise flat. A similar artifact can be seen on humans with low alpha-power but is invisible in humans with high alpha-power without trial-averaging. ***B***, Effect of a 0.1-s declination sweep of the horizonal magnetic component (inclination = +75°, total field = 35 µT, so horizontal component = 9.1 µT) rotating from NE to NW in the presence of a static, downward directed vertical magnetic field (33.8 µT; DecDn.CCW.N) and the reverse (DecDn.CW.N). This is a weaker electrical stimulus than used in ***A*** (only a 90° arc, a lower effective frequency of 2.5 Hz, and a quarter the field intensity). The cantaloupe shows only a weak artifact of <10 µV during the rotation. In most humans with high or low alpha-power, this artifact is hard to detect without extensive averaging. Artifacts of this sort are phase-locked to the stimulus and are easily removed using standard techniques for analyzing non-phase-locked power as noted in the EEG Methods section. Note that this human example shows an obvious drop in the alpha-power following the CCW rotation but not the CW rotation.

In active runs, an electromagnetic induction artifact occurred as a 10- to 40-μV fluctuation in the EEG signal during the 100-ms magnetic field rotation. The artifact was isolated and measured in EEG phantom experiments (presented in Materials and Methods, Part 2: details for replication and validation). Examples of single-trial, time-domain, bandpass-filtered (1–50 Hz) EEG traces at electrode Fz are shown in Figure 4. Figure 4*A* shows the artifact during the inclination rotation, measured from a cantaloupe and a human. The artifact is detectable in single trials from participants with low alpha-power (as shown), but difficult to see in participants with high alpha-power. Figure 4*B* shows the induction artifact during the declination rotation, which has smaller ∂**B**/∂*t* and produces a smaller artifact. The artifact is visible in the cantaloupe trace, but typically invisible in single-trial human EEG, especially in participants with high alpha-power (as shown). This induction artifact is similar to that observed in electrophysiological recordings from trout whenever magnetic field direction or intensity was suddenly changed in a square wave pattern (Walker et al., 1997). EEG artifacts induced by magnetic field shifts are induced in the presence of time-varying magnetic fields and disappear within a few milliseconds after the magnetic field shift (when ∂B/∂t = 0). This is true even in EEG studies involving transcranial magnetic stimulation where peak fields exceeding 2T are reached within 85 μs (resulting in 8 orders of magnitude greater ∂B/∂t than in our experiment). Artifacts in such concurrent TMS/EEG setups have been found to disappear within 5.6 ms (Veniero et al., 2009). Furthermore, the induction artifact is phase-locked like an event-related potential (ERP) and does not appear in analyses of non-phase-locked power, which we used in all subsequent statistical tests. Further discussion of electrical induction is in Materials and Methods, Part 2: details for replication and validation.

#### EEG data analysis

We used conventional methods of time/frequency decomposition (Morlet wavelet convolution) to compute post-stimulus power changes relative to a pre-stimulus baseline interval (−500 to −250 ms) over a 1- to 100-Hz frequency range. We focused on non-phase-locked power by subtracting the ERP in each condition from each trial of that condition before time/frequency decomposition. This is a well-known procedure for isolating non-phase-locked power and is useful for excluding the artifact from subsequent analyses (Cohen, 2014). Following the identification of alpha-band activity as a point of interest (detailed in Results), the following procedure was adopted to isolate alpha-activity in individuals. To compensate for known individual differences in peak resting alpha-frequency (8–12 Hz in our participant pool) and in the timing of alpha-wave responses following sensory stimulation, we identified individualized power change profiles using an automated search over an extended alpha-band of 6–14 Hz, 0–2 s post-stimulus. For each participant, power changes at electrode Fz were averaged over all trials, regardless of condition, to produce a single time/frequency map. In this cross-conditional average, the most negative time-frequency point was set as the location of the participant’s characteristic alpha-event-related-desynchronization (alpha-ERD). A window of 250 ms and 5-Hz bandwidth was automatically centered as nearly as possible on that point within the constraints of the overall search range. These search and window parameters were chosen based on typical alpha-ERD durations and bandwidths. The individualized window was used to test for significant differences between conditions. For each condition, power changes were averaged separately within the window, with trials subsampled and bootstrapped to equalize trial numbers across conditions. Outlier trials with extreme values of alpha-power (typically caused by movement artifacts or brief bursts of alpha-activity in an otherwise low-amplitude signal) in either the pre- or post-stimulus intervals were removed by an automated algorithm before averaging, according to a threshold of 1.5× the interquartile range of log alpha-power across all trials.

#### Software, data, and open access

Analyses were executed using automated turnkey scripts. Raw EEG data, the analysis code and documentation have been uploaded to the Caltech data repository and are available under Creative Commons Attribution-NonCommercial license (CC-BY-NC).

#### Human research protocol

Participants were 36 adult volunteers (24 male, 12 female) recruited from the Caltech population. This participant pool included persons of European, Asian, African and Native American descent. Ages ranged from 18 to 68 years. Each participant gave written informed consent of study procedures approved by the Caltech Institutional Review Board. All experiments were performed in accordance with relevant guidelines and regulations following NIH protocols for human experimentation, as reviewed and approved periodically by the Administrative Committee for the Protection of Human Subjects (Caltech IRB, protocols 13-0420, 17-0706, and 17-0734). All methods were carried out in accordance with relevant guidelines and regulations. Informed consent using forms approved by the Caltech Institutional Review Board was obtained from all subjects. No subjects under the age of 18 were used in these experiments.

### Part 2: details for replication and validation

#### Magnetic exposure facility

We constructed a six-sided Faraday cage shown in Figures 1, 2 out of aluminum, chosen because of (1) its high electrical conductivity, (2) low cost, and (3) lack of ferromagnetism. The basic structure of the cage is a rectangular 2.44 × 2.44 × 2.03-m frame made of aluminum rods, 1.3 × 1.3 cm square in cross-section (Fig. 2*A*). Each of the cage surfaces (walls, floor and ceiling) have four rods (two vertical and two horizontal) bounding the perimeter of each sheet. On the cage walls three vertical rods are spaced equally along the inside back of each surface, and on the floor and ceiling three horizontal rods are similarly spaced, forming an inwards-facing support frame. This frame provides a conductive chassis on which overlapping, 1-mm-thick aluminum sheets (2.44 m long and 0.91 m wide) were attached using self-threading aluminum screws at ∼0.60-m intervals with large overlaps between each sheet. In addition, we sealed the seams between separate aluminum panels with conductive aluminum tape. The access door for the cage is a sheet of aluminum that is fastened with a 2.4-m-long aluminum hinge on the East-facing wall such that it can swing away from the cage and provide an entrance/exit. Aluminum wool has been affixed around the perimeter of this entrance flap to provide a conductive seal when the flap is lowered (e.g., the cage is closed). Ventilation is provided via a ∼3-m-long, 15 cm in diameter flexible aluminum tube (Fig. 2*E*) that enters an upper corner of the room and is connected to a variable-speed ceiling-mounted fan set for a comfortable but quiet level of airflow. The end of the tube in contact with the Faraday cage is packed loosely with aluminum wool that allows air to pass and provides electrical screening. LED light strips (Fig. 2*H*) provide illumination for entrance and exit. These lights are powered by a contained lithium ion battery housed in an aluminum container attached at the top end of the Faraday cage, adjacent to the entrance of the ventilation air duct (Fig. 2*E*, red battery).

In all experiment sessions, power to the lights was switched off. A small USB-powered infrared camera and microphone assembly (Fig. 2*G*) mounted just inside the cage on the North wall allows audiovisual monitoring of participants inside the room. Instructions to the participants are given from a pair of speakers mounted outside the Faraday cage (Fig. 2*F*), controlled remotely by experimenters and electrically shorted by a computer-controlled TTL relay when not in use. Acoustic foam panels are attached to the vertical walls to dampen echoes within the chamber as well as to reduce the amplitude of external sound entering the chamber. To complete the Faraday shielding, we grounded the cage permanently at one corner with a 2.6 mm in diameter (10 AWG) copper wire connected to the copper plumbing in the sub-basement of the building. RMS noise measurements from the cage interior using a Schwarzbeck Mess Elektronik FMZB 1513 B-component active loop Rf antenna, a RIGOL DSA815/E-TG spectrum analyzer, and a Tektronix RSA503A signal analyzer indicated residual noise interference below 0.01 nT, in the frequency range from 9 kHz to 10 MHz.

Electrical cables entering the Faraday cage pass through a side gap in the aluminum ventilation duct and then through the aluminum wool. Rf interference is blocked further on all electrical cables entering the room using pairs of clip-on ferrite chokes (Fair-Rite material #75, composed of MnZn ferrite, designed for low-frequency EMI suppression, referred from here-on as ferrite chokes) and configured where possible using the paired, multiple-loop “pretty-good choke” configuration described by Counselman (2013; Fig. 2*I*). Inside the shielded space are located a three-axis set of square coils ∼2 m on edge following the Merritt et al. (1983) four-coil design (using the 59/25/25/59 coil winding ratio) that provides remarkably good spatial uniformity in the applied magnetic field (12 coils total, four each in the North/South, East/West, and Up/Down orientations; Fig. 2*A*). The coils are double-wrapped inside grounded aluminum U-channels following a design protocol that allows for full active-field and sham exposures (Kirschvink, 1992b); they were constructed by Magnetic Measurements, Ltd. This double-wrapped design gives a total coil winding count of 118/50/50/118 for all three-axes coil sets.

To provide a working floor isolated from direct contact with the coils, we suspended a layer of ∼2-cm-thick plywood sheets on a grid work of ∼10 × 10-cm-thick wooden beams that rested on the basal aluminum plate of the Faraday shield that are held together with brass screws. We covered this with a layer of polyester carpeting on top of which we placed a wooden platform chair for the participants (Fig. 2*B*). Non-magnetic bolts and screws were used to fasten the chair together, and a padded foam cushion was added for comfort. The chair is situated such that the head and upper torso of most participants fit well within the ∼1-m^3^ volume of highly uniform magnetic fields produced by the coil system (Kirschvink, 1992b) while keeping the participants a comfortable distance away from direct contact with the Merritt coils.

We suspended the three-axis probe of a fluxgate magnetometer (Applied Physics Systems model 520A) on a non-magnetic, carbon-fiber, telescoping camera rod suspended from the ceiling of the Faraday cage (Fig. 2*D*). This was lowered into the center of the coil system for initial calibration of field settings before experiments and then raised to the edge of the uniform field region to provide continuous recording of the magnetic field during experiments. Power cables for the coils and a data cable for the fluxgate sensor pass out of the Faraday cage through the ventilation shaft, through a series of large Rf chokes (Counselman, 2013), a ceiling utility chase in the adjacent hallway, along the wall of the control room, and finally down to the control hardware. The control hardware and computer are located ∼20 m away from the Faraday cage through two heavy wooden doors and across a hallway that serve as effective sound dampeners such that participants are unable to directly hear the experimenters or control equipment and the experimenters are unable to directly hear the participant.

In the remote-control room, three bipolar power amplifiers (Kepco model BOP-100-1MD) control the electric power to the coil systems (Fig. 2*J*) and operate in a mode where the output current is regulated proportional to the control voltage, thereby avoiding a drop in current (and magnetic field) should the coil resistance increase due to heating. Voltage levels for these are generated using a 10k samples per channel per second, 16-bit resolution, USB-controlled, analog output DAQ device (Measurement Computing Model USB 3101FS), controlled by the desktop PC. This same PC controls the DC power supply output levels, monitors and records the Cartesian orthogonal components from the fluxgate magnetometer, displays video of the participant (recordings of which are not preserved per IRB requirements), and is activated or shorted, via TTL lines, to the microphone/speaker communication system from the control room to the experimental chamber. As the experimenters cannot directly hear the participant and the participant cannot directly hear the experimenters, the microphone and speaker system are required (as per Caltech Institute Review Board guidelines) to ensure the safety and comfort of the participant as well as to pass instructions to the participant and answer participants’ questions before the start of a block of experiments. The three-axis magnet coil system can produce a magnetic vector of up to 100-μT intensity (roughly 2–3× the background strength in the lab) in any desired direction with a characteristic RL relaxation constant of 79–84 ms (inductance and resistance of the four coils in each axis vary slightly depending on the different coil-diameters for each of the three nested, double-wrapped coil-set axes). Active/sham mode was selected before each run via a set of double-pole-double-throw (DPDT) switches located near the DC power supplies. These DPDT switches are configured to swap the current direction flowing in one strand of the bifilar wire with respect to the other strand in each of the coil sets (Kirschvink, 1992b; Fig. 2*C*). Fluxgate magnetometer analog voltage levels were digitized and streamed to file via either a Measurement Computing USB 1608GX 8-channel (differential mode) analog input DAQ device, or a Measurement Computing USB 1616HS-2 multifunction A/D, D/A, DIO DAQ device connected to the controller desktop PC. Fluxgate analog voltage signal levels were sampled at 1024 or 512 Hz. Although the experimenter monitors the audio/video webcam stream of the participants continuously, as per Caltech IRB safety requirements, while they are in the shielded room, the control software disconnects the external speakers (in the room that houses the experimental Faraday cage and coils) and shorts them to electrical ground during all runs to prevent extraneous auditory cues from reaching the participants.

#### Experimental protocol

In the experiment, participants sat upright in the chair with their eyes closed and faced North (defined as 0° declination in our magnetic field coordinate reference frame). The experimental chamber was dark, quiet and isolated from the control room during runs. (Light levels within the experimental chamber during experimental runs were measured using a Konica-Minolta CS-100A luminance meter, which gave readings of zero, e.g., below 0.01 ± 2% cd/m^2^). Each run was ∼7 min long with up to eight runs in a ∼1-h session. The magnetic field was rotated over 100 ms every 2–3 s, with constant 2- or 3-s intertrial intervals in early experiments and pseudo-randomly varying 2- to 3-s intervals in later experiments. Participants were blind to active versus sham mode, trial sequence and trial timing. During sessions, auditory tones signaled the beginning and end of experiments and experimenters only communicated with participants once or twice per session to update the number of runs remaining. When time allowed, sham runs were matched to active runs using the same software settings. Sham runs are identical to active runs but are executed with the current direction switches set to anti-parallel. This resulted in no observable magnetic field changes throughout the duration of a sham run with the local, uniform, static field produced by the double-wrapped coil system in cancellation mode (Kirschvink, 1992b).

Two types of trial sequences were used: (1) a 127-trial Gold Sequence with 63 FIXED trials and 64 SWEEP trials evenly split between two rotations (32 each), and (2) various 150-trial pseudorandom sequences with 50 trials of each rotation interspersed with 50 FIXED trials to balance the number of trials in each of three conditions. All magnetic field parameters were held constant during FIXED trials, while magnetic field intensity was held constant during inclination or declination rotations. In inclination experiments (Fig. 3*A*), the vertical component of the magnetic field was rotated upwards and downwards between ±55°, ±60°, or ±75° (Inc.UP and Inc.DN, respectively); data collected from runs with each of these inclination values were collapsed into a single set representative of inclination rotations between steep angles. In each case, the horizontal component was steady at 0° declination (North; Inc.UP.N and Inc.DN.N). Two types of declination experiments were conducted, designed to test the quantum compass and electrical induction hypotheses. As the quantum compass can only determine the axis of the field and not polarity, we compared a pair of declination experiments in which the rotating vectors were swept down to the North (DecDn.N) and up to the South (DecUp.S), providing two symmetrical antiparallel datasets (Fig. 3*B*). In the DecDn.N experiments, the vertical component was held constant and downwards at +60° or +75°, while the horizontal component was rotated between NE (45°) and NW (315°), along a Northerly arc (DecDn.CW.N and DecDn.CCW.N). In DecUp.S experiments, the vertical component was held upwards at −60° or −75°, while the horizontal component was rotated between SW (225°) and SE (135°) along a Southerly arc (DecUp.CW.S and DecUp.CCW.S). Again, runs with differing inclination values were grouped together as datasets with steep downwards or steep upwards inclination. To test the induction hypothesis, we paired the DecDn.N sweeps with a similar set, DecUp.N (Fig. 3*C*). These two conditions only differ in the direction of the vertical field component; rotations were between NE and NW in both experiments (DecDn.CW.N, DecDn.CCW.N, DecUp.CW.N, and DecUp.CCW.N). Hence, any significant difference in the magnetosensory response eliminates induction as a mechanism.

#### EEG recording

EEG was recorded using a BioSemi ActiveTwo system with 64 electrodes following the International 10-20 System (Nuwer et al., 1998). Signals were sampled at 512 Hz with respect to CMS/DRL reference at low impedance <1 Ω and bandpass-filtered from 0.16 to 100 Hz. To reduce electrical artifacts induced by the time-varying magnetic field, EEG cables were bundled and twisted five times before plugging into a battery-powered BioSemi analog/digital conversion box. Digitized signals were transmitted over a 30 m, non-conductive, optical fiber cable to a BioSemi USB2 box located in the control room ∼20 m away where a desktop PC (separate from the experiment control system) acquired continuous EEG data using commercial ActiView software. EEG triggers signaling the onset of magnetic stimulation were inserted by the experiment control system by connecting a voltage timing signal (0–5 V) from the USB 3101FS analog output DAQ device. The timing signal was sent both to the Measurement Computing USB 1608GX (or USB 1616HS-2) analog input DAQ device, used to sample the magnetic field on the experiment control PC, and a spare DIO voltage input channel on the EEG system’s USB2 DAQ input box, which synchronized the EEG data from the optical cable with the triggers cued by the controlling desktop PC. This provided: (1) a precise timestamp in continuous EEG whenever electric currents were altered (or in the case of FIXED trials, when the electric currents could have been altered to sweep the magnetic field direction but were instead held constant) in the experimental chamber; and (2) a precise correlation (±2 ms, precision determined by the 512 samples per second digital input rate of the BioSemi USB2 box) between fluxgate and EEG data.

#### EEG analysis

Raw EEG data were extracted using EEGLAB toolbox for MATLAB (MATLAB, RRID:SCR_001622; EEGLAB, RRID:SCR_007292) and analyzed using custom MATLAB scripts. Trials were defined as 2- or 3-s epochs from −0.75 s pre-stimulus to +1.25 or +2.25 s post-stimulus, with a baseline interval from −0.5 s to −0.25 s pre-stimulus. Time/frequency decomposition was performed for each trial using Fast Fourier Transform (MATLAB function *fft*) and Morlet wavelet convolution on 100 linearly-spaced frequencies between 1 and 100 Hz. Average power in an extended alpha-band of 6–14 Hz was computed for the pre-stimulus and post-stimulus intervals of all trials, and a threshold of 1.5× the interquartile range was applied to identify trials with extreme values of log alpha-power. These trials were excluded from further analysis but retained in the data. After automated trial rejection, ERPs were computed for each condition and then subtracted from each trial of that condition to reduce the electrical induction artifact that appeared only during the 100-ms magnetic stimulation interval. This is an established procedure to remove phase-locked components such as sensory-evoked potentials from an EEG signal for subsequent analysis of non-phase-locked, time/frequency power representations. Non-phase-locked power was computed at midline frontal electrode Fz for each trial and then averaged and baseline-normalized for each condition to generate a time/frequency map from −0.25 s pre-stimulus to +1 s or +2 s post-stimulus and 1–100 Hz. To provide an estimate of overall alpha-power for each participant, power spectral density was computed using Welch’s method (MATLAB function *pwelch*) at 0.5-Hz frequency resolution (Welch, 1967).

From individual datasets, we extracted post-stimulus alpha-power to test for statistically significant differences among conditions at the group level. Because alpha-oscillations vary substantially across individuals in amplitude, frequency and stimulus-induced changes, an invariant time/frequency window would not capture stimulus-induced power changes in many participants. In our dataset, individual alpha-oscillations ranged in frequency (8- to 12-Hz peak frequency), and individual alpha-ERD responses started around +0.25 to +0.75 s post-stimulus. Thus, we quantified post-stimulus alpha-power within an automatically-adjusted time/frequency window for each dataset. First, non-phase-locked alpha-power between 6–14 Hz was averaged over all trials regardless of condition. Then, the most negative time/frequency point was automatically selected from the post-stimulus interval between 0 s and +1 or +2 s in this cross-conditional average. The selected point represented the maximum alpha-ERD in the average over all trials with no bias for any condition. A time/frequency window of 0.25 s and 5 Hz was centered (as nearly as possible given the limits of the search range) over this point to define an individualized timing and frequency of alpa-ERD for each dataset. Within the window, non-phase-locked alpha-power was averaged across trials and baseline-normalized for each condition, generating a value of alpha-ERD for each condition to be compared in statistical testing.

In early experiments, trial sequences were balanced with nearly equal numbers of FIXED (63) and SWEEP (64) trials, with an equal number of trials for each rotation (e.g., 32 Inc.DN and 32 Inc.UP trials). Later, trial sequences were designed to balance the number of FIXED trials with the number of trials of each rotation (e.g., 50 DecDn.FIXED, 50 DecDn.CCW, and 50 DecDn.CW trials). Alpha-ERD was computed over similar numbers of trials for each condition. For example, when comparing alpha-ERD in the FIXED versus CCW versus CW conditions of a declination experiment with 63 FIXED (32 CCW and 32 CW trials) 100 samplings of 32 trials were drawn from the pool of FIXED trials, alpha-ERD was averaged over the subset of trials in each sampling, and the average over all samplings was taken as the alpha-ERD of the FIXED condition. When comparing FIXED versus SWEEP conditions of an inclination experiment with 50 FIXED, 50 DN and 50 UP trials, 200 samplings of 25 trials were drawn from each of the DN and UP conditions and the average alpha-ERD over all samplings taken as the alpha-ERD of the SWEEP condition. Using this method, differences in experimental design were reduced, allowing statistical comparison of similar numbers of trials in each condition.

Three statistical tests were performed using average alpha-ERD: (1) Inc ANOVA (*N* = 29), (2) DecDn ANOVA (*N* = 26), (3) DecDn/DecUp ANOVA (*N* = 16). For the inclination experiment, data were collected in active and sham modes for 29 of 36 participants. Due to time limitations within EEG sessions, sham data could not be collected for every participant, so those participants without inclination sham data were excluded. A two-way repeated-measures ANOVA tested for the effects of inclination rotation (SWEEP vs FIXED) and magnetic stimulation (active vs sham) on alpha-ERD. *Post hoc* testing using the Tukey–Kramer method compared four conditions (Active-SWEEP, Active-FIXED, Sham-SWEEP and Sham-FIXED) for significant differences (Tukey, 1949).

For the DecDn experiment, data were collected from 26 participants in active mode. A one-way repeated-measures ANOVA tested for the effect of declination rotation (DecDn.CCW vs DecDn.CW vs DecDn.FIXED) with *post hoc* testing to compare these three conditions. For a subset of participants (*N* = 16 of 26), data were collected from both DecDn and DecUp experiments. The DecUp experiments were introduced in a later group to evaluate the quantum compass mechanism of magnetosensory transduction, as well as in a strongly-responding individual to test the less probable induction hypothesis, as shown in Movie 1. For tests of the quantum compass hypothesis, we used the DecDn/DecUp dataset. A two-way repeated-measures ANOVA tested for the effects of declination rotation (DecDn.CCW.N vs DecDn.CW.N vs DecUp.CCW.S vs DecUp.CW.S vs DecDn.FIXED.N vs DecUp.FIXED.S) and inclination direction (Inc.DN.N vs Inc.UP.S) on alpha-ERD; data from another strongly-responding individual is shown in Movie 2. *Post hoc* testing compared six conditions (DecDn.CCW.N, DecDn.CW.N, DecDn.FIXED.N, DecUp.CCW.S, DecUp.CW.S, and DecUp.FIXED.S).

Movie 1. Test of the electrical induction mechanism of magnetoreception using data from a participant with a strong, repeatable alpha-ERD magnetosensory response. Bottom row shows the DecDn.CCW.N, DecDn.CW.N and DecDn.FIXED.N conditions (64 trials per condition) of the DecDn.N experiment; top row shows the corresponding conditions for the DecUp.N experiment. Scalp topography changes from –0.25 s pre-stimulus to +1 s post-stimulus. The CCW rotation of a downwards-directed field (DecDn.CCW.N) caused a strong, repeatable alpha-ERD (lower left panel, *p* < 0.01 at Fz); weak alpha**-**power fluctuations observed in other conditions (DecDn.CW.N, DecDn.FIXED.N, DecUp.CW.N, DecUp.CCW.N, and DecUp.FIXED.N) were not consistent across multiple runs of the same experiment. If the magnetoreception mechanism is based on electrical induction, the same response should occur in conditions with identical ∂B/∂t (DecDn.CCW.N and DecUp.CCW.N), but the response was observed only in one of these conditions: a result that contradicts the predictions of the electrical induction hypothesis. 10.1523/ENEURO.0483-18.2019.movie.1

Movie 2. Test of the quantum compass mechanism of magnetoreception using data from another strongly-responding participant. Bottom versus top rows compare the DecDn.N and DecUp.S experiments in the CCW, CW, and FIXED conditions (DecDn.CCW.N, DecDn.CW.N, DecDn.FIXED.N, DecUp.CW.S, DecUp.CCW.S, and DecUp.FIXED.S with 100 trials per condition). The quantum compass is not sensitive to magnetic field polarity, so magnetosensory responses should be identical for the DecDn.CCW.N and DecUp.CCW.S rotations sharing the same axis. Our results contradict this prediction. A significant, repeatable alpha-ERD is only observed in the DecDn.CCW.N condition (lower left panel, *p* < 0.01 at Fz), with no strong, consistent effects in the DecUp.CCW.S condition (top left panel) or any other condition. 10.1523/ENEURO.0483-18.2019.movie.2

Within each group, certain participants responded strongly with large alpha-ERD while others lacked any response to the same rotations. To establish whether a response was consistent and repeatable, we tested individual datasets for significant post-stimulus power changes in time/frequency maps between 0 to +2 or +3 s post-stimulus and 1–100 Hz. For each dataset, 1000 permutations of condition labels over trials created a null distribution of post-stimulus power changes at each time/frequency point. The original time/frequency maps were compared with the null distributions to compute a p-value at each point. False discovery rate (FDR) correction for multiple comparisons was applied to highlight significant post-stimulus power changes at the *p* < 0.05 and *p* < 0.01 statistical thresholds (Benjamini and Hochberg, 1995).

#### Controlling for magnetomechanical artifacts

A question that arises in all studies of human perception is whether confounding artifacts in the experimental system produced the observed effects. The Sham experiments using double-wrapped, bonded coil systems controlled by remote computers and power supplies indicate that obvious artifacts such as resistive warming of the wires or magnetomechanical vibrations between adjacent wires are not responsible. In active mode, however, magnetic fields produced by the coils interact with each other with maximum torques occurring when the moment **u** of one coil set is orthogonal to the field **B** of another (torque = **u** × **B**). Hence, small torques on the coils might produce transient, sub-aural motion cues. Participants might detect these cues subconsciously although the coils are anchored to the Faraday cage at many points; the chair and floor assemblies are mechanically isolated from the coils; the experiments are run in total darkness, and the effective frequencies of change are all below 5 Hz and acting for only 0.1 s. No experimenters or participants ever claimed to perceive field rotations consciously even when the cage was illuminated and efforts were made to consciously detect the field rotations. Furthermore, the symmetry of the field rotations and the asymmetric nature of the results both argue strongly against this type of artifact. During the declination experiments, for example, the vertical component of the magnetic field is held constant while a constant-magnitude horizontal component is rotated 90° via the N/S and E/W coil axes. Hence, the torque pattern produced by DecDn.CCW.N rotations should be identical to that of the DecUp.CW.S rotations, yet these conditions yielded dramatically different results. We conclude that magnetomechanical artifacts are not responsible for the observed responses.

#### Testing for artifacts or perception from electrical induction

Another source of artifacts might be electrical eddy currents induced during field sweeps that might stimulate subsequent EEG brain activity in the head or perhaps in the skin or scalp adjacent to EEG sensors. Such artifacts would be hard to distinguish from a magnetoreceptive structure based on electrical induction. For example, the alpha-ERD effects might arise via some form of voltage-sensitive receptor in the scalp subconsciously activating sensory neurons and transmitting information to the brain for further processing. However, for any such electrical induction mechanism the Maxwell–Faraday law holds that the induced electric field **E** is related to the magnetic field vector, **B**(*t*), by:

1 ∇×E=−∂B(t)/∂t.

During a declination rotation, the field vector **B**(*t*) is given by: **B**(*t*) = **B**_V_ + **B**_H_(*t*), where **B**_V_ is the constant vertical field component, *t* is time, **B**_H_(*t*) is the rotating horizontal component, and the quantities in bold are vectors. Because the derivative of a constant is zero, the static vertical vector **B**_V_ has no effect, and the induced electrical effect depends only on the horizontally-rotating vector, **B**_H_(*t*):

2 $$\nabla \times E=-\partial B_{V}/\partial t-\partial B_{H}/\partial t=-\partial B_{H}(t)/\partial t.$$

In the induction test shown in Figure 3*C*, the sweeps of the horizontal component are identical, going along a 90° arc between NE and NW (DecDn.CCW.N and DecUp.CCW.N). The two trials differ only by the direction of the static vertical vector, **B**_V_, which is held in the downwards orientation for the bottom row of Movie 1 and upwards in the top row. Thus, divergent responses in these conditions cannot be explained based on electrical induction.

We also ran additional control experiments on “EEG phantoms,” which allow us to isolate the contribution of environmental noise and equipment artifacts. Typical setups range from simple resistor circuits to fresh human cadavers. We performed measurements on two commonly-used EEG phantoms: a bucket of saline, and a cantaloupe. From these controls, we isolated the electrical effects induced by magnetic field rotations. The induced effects were similar to the artifact observed in human participants during the 100-ms magnetic stimulation interval, and noted on Figure 4. In cantaloupe and in the water-bucket controls, no alpha-ERD responses were observed in active or sham modes suggesting that a brain is required to produce a magnetosensory response downstream of any induction artifacts in the EEG signal.

### Online content

All digital data are available at https://doi.org/10.22002/d1.930 and https://doi.org/10.22002/d1.931, including MATLAB scripts used for the automatic data analysis.

## Results

### Neural response to geomagnetic stimuli

**Figure 5.**
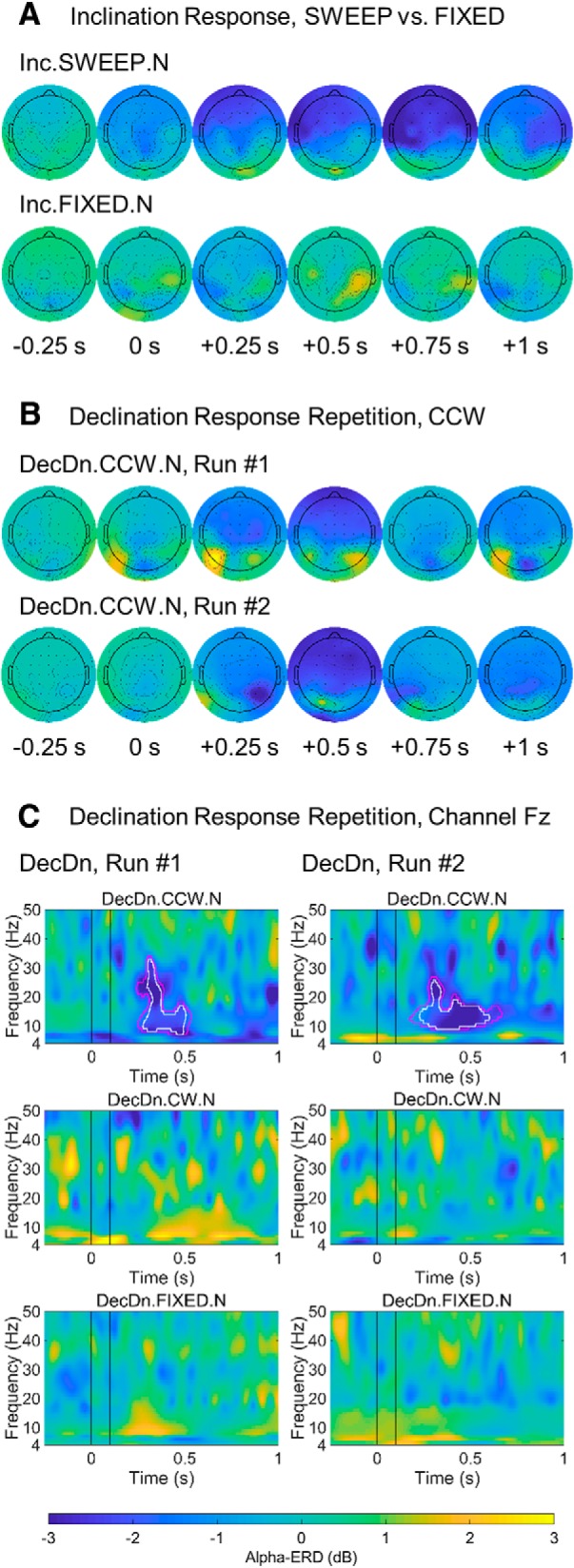
Alpha-ERD as a neural response to magnetic field rotation. Post-stimulus power changes (dB) from a pre-stimulus baseline (−500 to −250 ms) plotted according to the ±3-dB color bar at bottom. ***A***, Scalp topography of the alpha-ERD response in an inclination experiment, showing alpha-power at select time points before and after field rotation at 0 s. Alpha-ERD (deep blue) was observed in SWEEP (top row), but not FIXED (bottom row), trials. ***B***, Scalp topography of the alpha-ERD response for two runs of the declination experiment, tested six months apart in a different strongly-responding participant. DecDn.CCW.N condition is shown. In both runs, the response peaked around +500 ms post-stimulus and was widespread over frontal/central electrodes, demonstrating a stable and reproducible response pattern. ***C***, Time-frequency maps at electrode Fz for the same runs shown in ***B***. Black vertical lines indicate the 0- to 100-ms field rotation interval. Pink/white outlines indicate significant alpha-ERD at the *p* < 0.05 and *p* < 0.01 statistical thresholds, respectively. Separate runs shown side by side. Significant alpha-ERD was observed following downwards-directed counterclockwise rotations (outlines in top row) with no other power changes reaching significance. Significant power changes appear with similar timing and bandwidth, while activity outside the alpha-ERD response and activity in other conditions is inconsistent across runs.

In initial observations, several participants (residing in the Northern Hemisphere) displayed striking patterns of neural activity following magnetic stimulation, with strong decreases in EEG alpha-power in response to two particular field rotations: (1) inclination SWEEP trials (Inc.UP.N and Inc.DN.N), in which the magnetic vector rotated either down or up (e.g., rotating a downwards pointed field vector up to an upwards pointed vector, or vice versa; Fig. 3*A*, red and blue arrows); and (2) DecDn.CCW.N trials, in which magnetic field declination rotated counterclockwise while inclination was held downwards (as in the Northern Hemisphere; Fig. 3*B*, solid red arrow). Alpha-power began to drop from pre-stimulus baseline levels as early as ∼100 ms after magnetic stimulation, decreasing by as much as ∼50% over several hundred milliseconds, then recovering to baseline by ∼1 s post-stimulus. Figure 4*B* shows a sample EEG voltage trace that contains such a drop in alpha-power. The time-frequency power maps in Figure 5 are cross-trial averages and show how the spectral power contained in the EEG trace changed across time. Drops in power are depicted in a deep blue color. Scalp topography was bilateral and widespread, centered over frontal/central electrodes, including midline frontal electrode Fz when referenced to CPz. Figure 5*A* shows the whole-brain response pattern to inclination sweeps and control trials (Inc.SWEEP.N and Inc.FIXED.N) of one of the responsive participants, with the alpha-ERD exhibited in the SWEEP but not FIXED trials. Similarly, Figure 5*B*,*C* shows the declination responses of a different participant on two separate runs (labeled runs 1 and 2) six months apart. Response timing, bandwidth and topography of the alpha-ERD in the CCW sweeps, with negative FIXED controls, were replicated across runs, indicating a repeatable signature of magnetosensory processing in humans. After experimental sessions, participants reported that they could not discern when or if any magnetic field changes had occurred.

The alpha-rhythm is the dominant human brain oscillation in the resting state when a person is not processing any specific stimulus or performing any specific task (Klimesch, 1999). Neurons engaged in this internal rhythm produce 8- to 13-Hz alpha-waves that are measurable by EEG. Individuals vary widely in the amplitude of the resting alpha-rhythm. When an external stimulus is suddenly introduced and processed by the brain, the alpha-rhythm generally decreases in amplitude compared with a pre-stimulus baseline. (Pfurtscheller et al., 1994; Klimesch, 1999; Hartmann et al., 2012). This EEG phenomenon, termed alpha-ERD, has been widely observed during perceptual and cognitive processing across visual, auditory and somatosensory modalities (Peng et al., 2012). Alpha-ERD may reflect the recruitment of neurons for processing incoming sensory information and is thus a generalized signature for a shift of neuronal activity from the internal resting rhythm to external engagement with sensory or task-related processing (Pfurtscheller and Lopes da Silva, 1999). Individuals also vary in the strength of alpha-ERD; those with high resting-state or pre-stimulus alpha-power tend to show strong alpha-ERDs following sensory stimulation, while those with low alpha-power have little or no response in the alpha-band (Klimesch, 1999).

Based on early observations, we formed the hypothesis that sensory transduction of geomagnetic stimuli could be detectable as alpha-ERD in response to field rotations, e.g., the magnetic field rotation would be the external stimulus, and the alpha-ERD would be the signature of the brain beginning to process sensory data from this stimulus. This hypothesis was tested at the group level in data collected from 29 participants in the inclination rotation conditions (Fig. 3*A*) and 26 participants in the declination rotation conditions (Fig. 3*B*, solid arrows).

**Figure 6.**
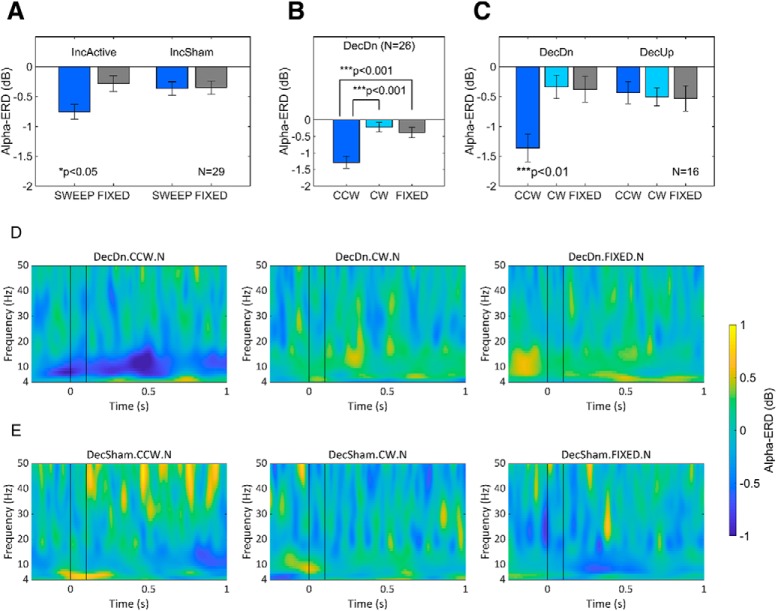
Group results from repeated-measures ANOVA for the effects of geomagnetic stimulation on post-stimulus alpha-power. ***A***, Average alpha-ERD (dB) at electrode Fz in the SWEEP and FIXED conditions of inclination experiments run in active or sham mode. Two-way ANOVA showed an interaction (*p* < 0.05, *N* = 29) of inclination rotation (SWEEP vs FIXED) and magnetic stimulation (active vs sham). According to *post hoc* testing, only inclination sweeps in active mode produced alpha-ERD above background fluctuations in FIXED trials (*p* < 0.01) or sham mode (*p* < 0.05). ***B***, Average alpha-ERD (dB) at electrode Fz in the declination experiment with inclination held downwards (DecDn). One-way ANOVA showed a significant main effect of declination rotation (*p* < 0.001, *N* = 26). The downwards-directed counterclockwise rotation (DecDn.CCW.N) produced significantly different effects from both the corresponding clockwise rotation (DecDn.CW.N, *p* < 0.001) and the FIXED control condition (DecDn.FIXED.N, *p* < 0.001). ***C***, Comparison of the declination rotations with inclination held downwards (DecDn) or upwards (DecUp) in a subset (*N* = 16 of 26) of participants run in both experiments. Two-way ANOVA showed a significant interaction (*p* < 0.01) of declination rotation (CCW vs CW vs FIXED) and inclination direction (Dn vs Up). *Post hoc* testing showed significant differences (*p* < 0.01) between the DecDn.CCW.N condition and every other condition, none of which were distinct from any other. This is a direct test and rejection of the quantum compass hypothesis. ***D***, Grand average of time-frequency power changes across the 26 participants in the DecDn experiment from ***B***. Black vertical lines indicate the 0- to 100-ms field rotation interval. A post-stimulus drop in alpha-power was observed only following the downwards-directed counterclockwise rotation (left panel). Wider spread of desynchronization reflects interindividual variation. Convolution involved in time/frequency analyses causes the early responses of a few participants to appear spread into the pre-stimulus interval. ***E***, Grand average of time-frequency power changes across the 18 participants with sham data in the declination experiments; no significant power changes were observed.

For inclination experiments, we collected data from matched active and sham runs (*N* = 29 of 36; seven participants were excluded due to time limits that prevented the collection of sham data). We tested for the effects of inclination rotation (SWEEP vs FIXED) and magnetic stimulation (active vs sham) using a two-way repeated-measures ANOVA. We found a significant interaction of inclination rotation and magnetic stimulation (*p* < 0.05). *Post hoc* comparison of the four experimental conditions (active-SWEEP, active-FIXED, sham-SWEEP, sham-FIXED) revealed significant differences between active-SWEEP and all other conditions (*p* < 0.05). Downwards/upwards rotations of magnetic field inclination produced an alpha-ERD ∼2× greater than background fluctuations in the FIXED control condition and all the sham conditions. Results are summarized in Table 1 and Figure 6*A*.

**Table 1.** Group results from two-way, repeated-measures ANOVA for the effects of inclination rotation × magnetic stimulation on post-stimulus alpha-power

ANOVA 1. Effects of inclination rotation and magnetic stimulation on post-stimulus alpha-power			
Two-way repeated measures ANOVA (*N* = 29) Inclination rotation × magnetic stimulation	*F*	*p*	η_p_^2^
Main effect of inclination rotation (SWEEP vs FIXED)	3.26	0.08	0.19
Main effect of magnetic stimulation (active vs sham)	2.47	0.13	0.09
Inclination rotation × magnetic stimulation (interaction)	5.67	0.02*	0.17

ANOVA 1 shows a significant interaction of inclination rotation (SWEEP vs FIXED) and magnetic stimulation (active vs sham) in the inclination experiments. Based on *post hoc* testing, alpha-ERD was significantly greater in SWEEP trials in active mode, compared with all other conditions (*p* < 0.05). In this table, *F* is the *F*-ratio statistic, *p* the probability value, and η_p_^2^ the partial η^2^ value from the ANOVA.

In declination experiments (Fig. 6*B*), we observed a strikingly asymmetric response to the clockwise (DecDn.CW.N) and counterclockwise (DecDn.CCW.N) rotations of a downwards-directed field sweeping between Northeast and Northwest. Alpha-ERD was ∼3× greater after counterclockwise than after clockwise rotations, the latter producing alpha-power changes indistinguishable from background fluctuations in the FIXED control condition. Over the participant pool (*N* = 26 of 26 who were run in this experiment), we ran a one-way repeated-measures ANOVA with three conditions (DecDn.CCW.N, DecDn.CW.N and DecDn.FIXED.N) to find a highly significant effect of declination rotation (*p* < 0.001; Table 2). As indicated in Figure 6*B*, the counterclockwise rotation elicited a significantly different response from both the clockwise rotation (*p* < 0.001) and FIXED control (*p* < 0.001). Figure 6*D* shows the stimulus-locked grand average across all participants for each condition; an alpha-ERD is observed only for counterclockwise rotations of a downwards-directed field (left panel). Sham data were available for 18 of 26 participants in the declination experiments; no major changes in post-stimulus power were observed in any of the sham conditions (Fig. 6*E*).

**Table 2.** Group results from one-way, repeated-measures ANOVA for the effects of declination rotation at downwards inclination on post-stimulus alpha-power

ANOVA 2. Effects of declination rotation at downwards inclination on post-stimulus alpha-power			
One-way repeated measures ANOVA (*N* = 26)	*F*	*P*	η_p_^2^
Main effect of declination rotation (CCW vs CW vs FIXED)	13.09	0.00003***	0.34

ANOVA 2 shows a significant main effect of declination rotation when the inclination is static and downwards as in the Northern Hemisphere. Based on *post hoc* testing, alpha-ERD was significantly greater in CCW trials than in CW or FIXED trials (*p* < 0.001). *F* is the *F*-ratio statistic, *p* the probability value, and η_p_^2^ the partial η^2^ value from the ANOVA.

The asymmetric declination response provided a starting point for evaluating potential mechanisms of magnetosensory transduction, particularly the quantum compass hypothesis, which has received much attention in recent years (Ritz et al., 2000; Hore and Mouritsen, 2016). Because the quantum compass cannot distinguish polarity, we conducted additional declination rotation experiments in which the fields were axially identical to those in the preceding DecDn experiments, except with reversed polarity (Fig. 3*B*, reversed polarity rotations shown as dashed arrows). In the additional DecUp conditions, Magnetic North pointed to Geographic South and up rather than Geographic North and down, and the upwards-directed field rotated clockwise (DecUp.CW.S) or counterclockwise (DecUp.CCW.S) between SE and SW. In later testing, we ran 16 participants in both the DecDn and DecUp experiments to determine the effects of declination rotation and inclination direction in a two-way repeated measures ANOVA with six conditions (DecDn.CCW.N, DecDn.CW.N, DecDn.FIXED.N, DecUp.CCW.S, DecUp.CW.S, and DecUp.FIXED.S). A significant interaction of declination rotation and inclination direction (*p* < 0.01) was found (Fig. 6*C*; Table 3). DecDn.CCW.N was significantly different from all other conditions (*p* < 0.01), none of which differed from any other. Thus, counterclockwise rotations of a downwards-directed field were processed differently in the human brain from the same rotations of a field of opposite polarity. These results contradict the quantum compass hypothesis, as explained below in Biophysical mechanisms.

**Table 3.** Group results from two-way, repeated-measures ANOVA for the effects of declination rotation × inclination direction on post-stimulus alpha-power

ANOVA 3. Effects of declination rotation and inclination direction on post-stimulus alpha-power			
Two-way repeated measures ANOVA (*N* = 16) Declination rotation × inclination direction	*F*	*p*	η_p_^2^
Main effect of declination rotation (CCW vs CW vs FIXED)	3.77	0.03*	0.24
Main effect of inclination direction (Dn vs Up)	0.89	0.36	0.06
Declination rotation × inclination direction (interaction)	6.49	0.004***	0.30

ANOVA 3 shows a significant interaction of declination rotation and inclination direction in declination experiments designed to test the “quantum compass” mechanism of magnetoreception. A significant alpha-ERD difference (*p* < 0.05) between counterclockwise down (DecDn.CCW.N) and counterclockwise up (DecUp.CCW.S) argues against this hypothesis in humans. *F* is the *F*-ratio statistic, *p* the probability value, and η_p_^2^ the partial η^2^ value from the ANOVA.

**Figure 7.**
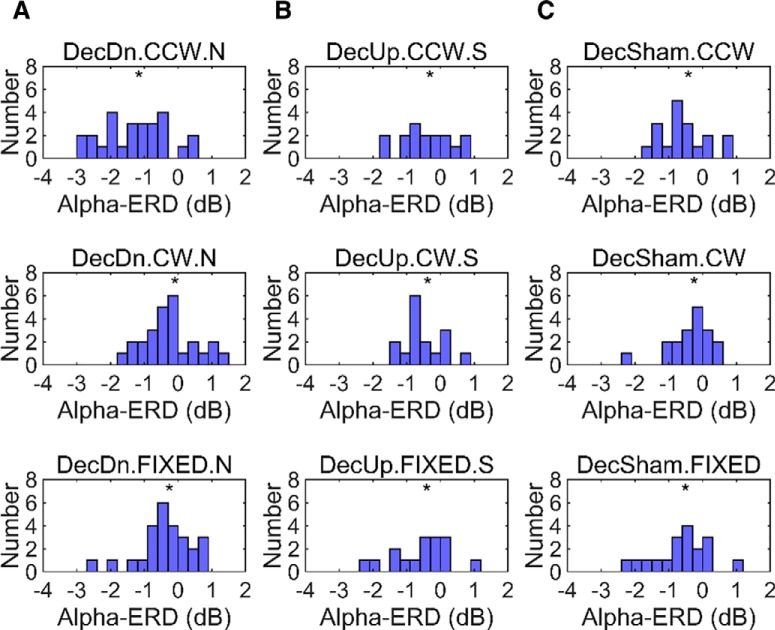
Histogram of alpha-ERD responses over all participants. The panels show the histogram of individual responses for each condition. Frequency is given in number of participants. Because we looked for a drop in alpha**-**power following magnetic stimulation, the histograms are shifted toward negative values in all conditions. ***A***, Standard DecDn experiment (*N* = 26). The CCW condition shows the most negative average in a continuous distribution of participant responses with the most participants having a >2**-**dB response. ***B***, DecUp experiment (*N* = 16). No significant magnetosensory response was observed in any condition, and no clear difference is apparent between the three distributions. ***C***, Sham declination experiment (*N* = 18). No significant magnetosensory response was observed in any condition, and no clear difference is apparent between the three distributions.

From previous EEG studies of alpha-oscillations in human cognition, the strength of alpha-ERD is known to vary substantially across individuals (Pfurtscheller et al., 1994; Klimesch et al., 1998; Klimesch, 1999). In agreement with this, we observed a wide range of alpha-ERD responses in our participants as well. Some participants showed large drops in alpha-power up to ∼60% from pre-stimulus baseline, while others were unresponsive with little change in post-stimulus power at any frequency. Histograms of these responses are provided in Figure 7.

**Figure 8.**
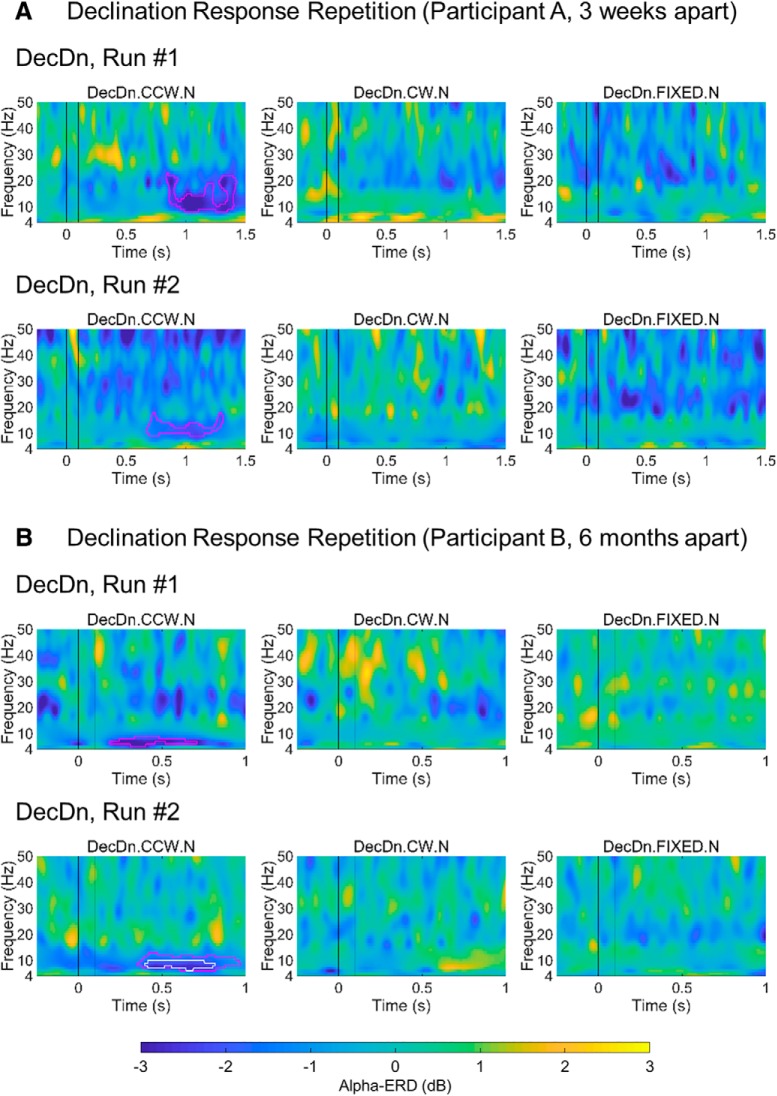
Repeated results from two strongly-responding participants. In both ***A***, ***B***, participants were tested weeks or months apart under the same conditions (run 1 and run 2). Time/frequency maps show similar timing and bandwidth of significant alpha**-**power changes (blue clusters in outlines) after counterclockwise rotation, while activity outside the alpha-ERD response, and activity in other conditions is inconsistent across runs. Pink/white outlines indicate significance at the *p* < 0.05 and *p* < 0.01 thresholds. The participant in ***A*** had an alpha**-**peak frequency at >11 Hz and a lower-frequency alpha-ERD response. The participant in ***B*** had an alpha**-**peak frequency <9 Hz and a higher-frequency alpha-ERD response. Minor power fluctuations in the other conditions or in different frequency bands were not repeated across runs, indicating that only the alpha-ERD was a repeatable signature of magnetosensory processing.

To confirm that the variability across the dataset was due to characteristic differences between individuals rather than general variability in the measurement or the phenomenon, we retested the strongly-responding participants to see whether their responses were stable across sessions. Using permutation testing with FDR correction at the *p* < 0.05 and *p* < 0.01 statistical thresholds, we identified participants who exhibited alpha-ERD that reached significance at the individual level and tested them (*N* = 4) again weeks or months later. An example of separate runs on the same participant is shown in Figure 5*B*,*C*, and further data series are shown in the Figure 8. Each participant replicated their results with similar response tuning, timing and topography, providing greater confidence that the observed effect was specific for the magnetic stimulus in the brain of that individual. While the functional difference between strongly and weakly responding individuals is unclear, the identification of strongly responding individuals gives us the opportunity to conduct more focused tests directed at deriving the biophysical characteristics of the transduction mechanism.

### Biophysical mechanisms

Three major biophysical transduction hypotheses have been considered extensively for magnetoreception in animals: (1) various forms of electrical induction (Yeagley, 1947; Kalmijn, 1981; Rosenblum et al., 1985), (2) a chemical/quantum compass involving hyperfine interactions with a photoactive pigment (Schulten, 1982) like cryptochrome (Ritz et al., 2000; Hore and Mouritsen, 2016), and (3) specialized organelles based on biologically-precipitated magnetite similar to those in magnetotactic microorganisms (Kirschvink and Gould, 1981). We designed the declination experiments described above to test these hypotheses.

#### Electrical induction

According to the Maxwell–Faraday law (∇ × **E** = -∂**B**/∂*t*), electrical induction depends only on the component of the magnetic field that is changing with time (∂**B**/∂*t*). In our declination experiments, this corresponds to the horizontal component that is being rotated. The vertical component is held constant and therefore does not contribute to electrical induction. Thus, we compared brain responses to two matched conditions, where the declination rotations were identical, but the static vertical components were opposite (Fig. 3*C*). A transduction mechanism based on electrical induction would respond identically to these two conditions. Movie 1 shows the alpha-ERD magnetosensory response of one strongly-responding individual to these two stimulus types. In the top row, the static component was pointing upwards, and in the bottom row, the static field was pointing downwards. In the DecDn.CCW.N condition (lower left panel), the alpha-ERD (deep blue patch) starts in the right parietal region almost immediately after magnetic stimulation and spreads over the scalp to most recording sites. This large, prolonged and significant bilateral desynchronization (*p* < 0.01 at Fz) occurs only in this condition with only shorter, weaker and more localized background fluctuations in the other conditions (n.s. at Fz). No alpha-ERD was observed following any upwards-directed field rotation (DecUp.CCW.N and DecUp.CW.N, top left and middle panels), in contrast to the strong response in the DecDn.CCW.N condition.

Looking at data across all of our experiments (on people from the Northern Hemisphere) no participant produced alpha-ERD responses to rotations with a static vertical-upwards magnetic field (found naturally in the Southern Hemisphere). This demonstrates that the observed, non-phase-locked alpha-ERD in participants is not an artifact, as the alpha-ERD discriminates between geomagnetic field rotations that are identical in their dynamic component but differ only in their static components. This level of discrimination demands that some form of sensory transduction and neural processing of that transduced signal must be occurring in the human participants.

These tests indicate that electrical induction mechanisms cannot account for the neural response. This analysis also rules out an electrical artifact of induced current loops from the scalp electrodes, as any current induced in the loops would also be identical across the matched runs. Our results are also consistent with many previous biophysical analyses, which argue that electrical induction would be a poor transduction mechanism for terrestrial animals, as the induced fields are too low to work reliably without large, specialized anatomic structures that would have been identified long ago (Yeagley, 1947; Rosenblum et al., 1985). Other potential confounding artifacts were discussed in Materials and Methods, Part 2: details for replication and validation.

#### Quantum compass

From basic physical principles, a transduction mechanism based on quantum effects can be sensitive to the axis of the geomagnetic field but not the polarity (Schulten, 1982; Ritz et al., 2000). In the most popular version of this theory, a photosensitive molecule like cryptochrome absorbs a blue photon, producing a pair of free radicals that can transition between a singlet and triplet state with the transition frequency depending on the local magnetic field. The axis of the magnetic field, but not the polarity, could then be monitored by differential reaction rates from the singlet versus triplet products.

This polarity insensitivity, shared by all quantum-based magnetotransduction theories, is inconsistent with the group level test of the quantum compass presented above. The data (Table 3; Fig. 6*C*, dark blue bars) showed clearly distinct responses depending on polarity. We additionally verified this pattern of results at the individual level. Movie 2 shows the alpha-ERD magnetosensory response in another strongly-responding individual. Only the DecDn.CCW.N rotation (lower left panel) yields a significant alpha-ERD (*p* < 0.01 at Fz). Lack of a significant response in the axially identical DecUp.CCW.S condition indicates that the human magnetosensory response is sensitive to polarity.

On the surface, it can seem that non-polar inputs can support polarity-dependent behavior by supplementing with other sensory cues such as gravity. Birds and some other animals display a magnetic inclination compass that identifies the steepest angle of magnetic field dip with respect to gravity (Wiltschko, 1972; Wiltschko and Wiltschko, 1995a). In the context of the Earth’s magnetic field, this non-polar cue allows a bird to identify the direction of the closest pole but does not allow it to identify whether it is the North or the South. This behavioral strategy could not distinguish between the antipodal (vector opposite) fields used in our biophysical test of polarity sensitivity. If we create a field with magnetic north down and to the front, the bird might correctly identify North as forward. However, if we point magnetic north up and to the back, that bird would continue to identify North as forward because that is the direction of maximum dip. In the end, magnetism and gravity are distinct, non-interacting forces of nature, and so magnetic polarity information cannot be extracted from gravity.

In our experiment, the initial magnetic transduction mechanism must be sensitive to polarity to give rise to a neural response that is sensitive to polarity. If polarity information is not present initially from a magnetic transducer, it cannot be recovered by adding information from other sensory modalities. As an illustration, if we gave our participants a compass with a needle that did not have its North tip marked, they could not distinguish the polarity of an applied magnetic field even if we gave them a gravity pendulum or any other non-magnetic sensor. This means that a quantum compass-based mechanism cannot account for the alpha-ERD response we observe in humans.

## Discussion

### Response selectivity

The selectivity of brain responses for specific magnetic field directions and rotations may be explained by tuning of neural activity to ecologically relevant values. Such tuning is well known in marine turtles in the central Atlantic Ocean, where small increases in the local geomagnetic inclination or intensity (that indicate the animals are drifting too far North and are approaching the Gulf Stream currents) trigger abrupt shifts in swimming direction, thereby preventing them from being washed away from their home in the Sargasso Sea (Light et al., 1993; Lohmann and Lohmann, 1996; Lohmann et al., 2001). Some migratory birds are also known to stop responding to the magnetic direction if the ambient field intensity is shifted more than ∼25% away from local ambient values (Wiltschko, 1972), which would stop them from using this compass over geomagnetic anomalies. From our human experiments to date, we suspect that alpha-ERD occurs in our participants mainly in response to geomagnetic fields that reflect something close to “normal” in our Northern Hemisphere locale, where the North-seeking field vector tilts downwards. This would explain why field rotations with a static upwards component produced little response in Northern Hemisphere participants. Conducting similar experiments on participants born and raised in other geographic regions (such as in the Southern Hemisphere or on the Geomagnetic Equator) could test this hypothesis.

Another question vis-à-vis response selectivity is why downwards-directed CCW (DecDn.CCW.N), but not CW (DecDn.CW.N), rotations elicited alpha-ERD. The bias could arise at various levels, either at the receptor or during neural processing. The structure and function of the magnetoreceptor cells are unknown, but biological structures exhibit chirality (right or left handedness) at many spatial scales, from individual amino acids to folded protein assemblies to multicellular structures. If such mirror asymmetries exist in the macromolecular complex interfacing with magnetite, they could favor the transduction of one stimulus over its opposite. Alternatively, higher-level cognitive processes could tune the neural response toward counterclockwise rotations without any bias at the receptor level. As of this writing, we cannot rule out the possibility that some fraction of humans may have a CW response under this or other experimental paradigms, just as some humans are left- instead of right-handed. We also cannot rule out the existence of a separate neural response to CW rotations that is not reflected in the alpha-ERD signature that we assay here.

The functional significance of the divergent responses to CW and CCW is also unclear. It may simply arise as a by-product during the evolution and development of more ecologically relevant mirror asymmetries (such as North-up vs North-down). It may also be that the alpha-ERD response reflects non-directional information, such as a warning of geomagnetic anomalies, which can expose a navigating animal to sudden shifts of the magnetic field comparable to those used in our experiments. Entering and exiting local anomalies exposes animals to opposite field shifts, and sensitivity to one of the paired shift directions is sufficient to detect the anomaly. For example, volcanic or igneous terranes are prone to fields of such anomalies due to remagnetization by lightning strikes (Carporzen et al., 2012). An animal moving through magnetic features of this sort will receive a series of warning signals against using the magnetic field for long-range navigation. Future experiments could test this speculation by sweeping field intensity through values matching those of lightning-strike and other anomalies to check for asymmetric patterns of alpha-ERD.

A final question is whether the response asymmetry occurs only in passive experiments when participants experience magnetic stimulation without attempting to make use of the information. Neural processing in other sensory domains is known to vary in its tuning depending on the organisms’ behavioral or attentive state (Fontanini and Katz, 2008). Behavioral tasks, such as judging the direction or rotation of the field with EEG recording could be used to explore the magnetosensory system in more detail and to see whether response selectivity is affected.

### General discussion

As noted above, many past attempts have been made to test for the presence of human magnetoreception using behavioral assays, but the results were inconclusive. To avoid the cognitive and behavioral artifacts inherent in testing weak or subliminal sensory responses, we decided to use EEG techniques to see directly whether or not the human brain has passive responses to magnetic field changes. Our results indicate that human brains are indeed collecting and selectively processing directional input from magnetic field receptors. These give rise to a brain response that is selective for field direction and rotation with a pattern of neural activity that is measurable at the group level and repeatable in strongly-responding individuals. The selectivity of the response favored ecologically valid stimuli, distinguishing between rotations of otherwise equal speeds and magnitudes. This indicates that the effect is due to a biologically tuned mechanism rather than some generic physical influence. Such neural activity is a necessary prerequisite for any subsequent behavioral expression of magnetoreception, and it represents a starting point for testing whether such an expression exists.

The fact that alpha-ERD is elicited in a specific and sharply delineated pattern allows us to make inferences regarding the biophysical mechanisms of signal transduction. Notably, the alpha-ERD response differentiated clearly between sets of stimuli differing only by their static or polar components. Electrical induction, electrical artifacts and quantum compass mechanisms are totally insensitive to these components and cannot account for the selectivity of the brain responses we recorded. In contrast, ferromagnetic mechanisms can be highly sensitive to both static and polar field components and could distinguish our test stimuli with different responses. In the simplest form, the torque (= **u** × **B**) from a string of magnetite crystals (a “magnetosome chain” like those in the magnetotactic bacteria) could act to open and close trans-membrane ion channels. Several biophysical analyses have shown this is a most plausible mechanism (Kirschvink, 1992a; Winklhofer and Kirschvink, 2010). Finally, magnetite-based mechanisms for navigation have been characterized in animals through neurophysiological (Walker et al., 1997), histologic (Diebel et al., 2000), and pulse-remagnetization studies (Kirschvink and Kobayashi-Kirschvink, 1991; Wiltschko et al., 1994, 1998, 2002, 2007, 2009; Wiltschko and Wiltschko, 1995b; Beason et al., 1997; Munro et al., 1997a, b; Irwin and Lohmann, 2005; Holland et al., 2008; Holland, 2010; Holland and Helm, 2013; Ernst and Lohmann, 2016), and biogenic magnetite has been found in human tissues (Kirschvink et al., 1992; Dunn et al., 1995; Kobayashi and Kirschvink, 1995; Schultheiss-Grassi et al., 1999; Maher et al., 2016; Gilder et al., 2018).

These data argue strongly for a geomagnetic transduction mechanism similar to those in numerous migratory and homing animals. Single-domain ferromagnetic particles such as magnetite are directly responsive to both time-varying and static magnetic fields and are sensitive to field polarity. At the cellular level, the magnetomechanical interaction between ferromagnetic particles and the geomagnetic field is well above thermal noise (Kirschvink and Gould, 1981; Kirschvink et al., 2010), stronger by several orders of magnitude in some cases (Eder et al., 2012). In many animals, magnetite-based transduction mechanisms have been found and shown to be necessary for navigational behaviors, through neurophysiological and histologic studies (Walker et al., 1997; Diebel et al., 2000). A natural extension of this study would be to apply the pulse-remagnetization methods used in animals to directly test for a ferromagnetic transduction element in humans. In these experiments, a brief magnetic pulse causes the magnetic polarity of the single-domain magnetite crystals to flip. Following this treatment, the physiologic and behavioral responses to the geomagnetic field are expected to switch polarity. These experiments could provide measurements of the microscopic coercivity of the magnetite crystals involved and hence make predictions about the physical size and shape of the crystals, and perhaps their physiologic location.

At this point, our observed reduction in alpha-band power is a clear neural signature for cortical processing of the geomagnetic stimulus, but its functional significance is unknown. In form, the activity is an alpha-ERD response resembling those found in other EEG investigations of sensory and cognitive processing. However, the alpha-ERD responses found in literature take on a range of different spatiotemporal forms and are associated with a variety of functions. It is likely that the alpha-ERD seen here reflects the sudden recruitment of neural processing resources, as this is a finding common across studies. But more research will be needed to see whether and how it relates more specifically to previously studied processes such as memory access or recruitment of attentional resources.

Further, an alpha-ERD response is a fairly broad signature of neural activity: an obvious feature of a complex array of neural processes. A host of upstream and downstream processes need to be investigated to reveal the network of responses and the information they encode. Responses independent from the alpha-ERD signature may also emerge, and those responses might show different selectivity patterns and reflect stimulus features not revealed in this study. Does human magnetoreceptive processing reflect a full representation of navigational space? Does it contain certain warning signals regarding magnetic abnormalities? Or have some aspects degenerated from the ancestral system? For now, alpha-ERD remains a blank signature for a wider, unexplored range of magnetoreceptive processing.

Our experimental methodology differs from previous studies in a number of ways that may explain their negative or equivocal outcomes. First, previous EEG studies (Boorman et al., 1999; Sastre et al., 2002) often used stimuli outside the environmental range. While sensory systems generally display response specificity and neural tuning to the local environment (Block, 1992), they can be less responsive or un-responsive to unnatural stimuli. For example, in four of seven conditions from Sastre et al. (2002; A, B, C, and D), the field intensities used (90 μT) were twice as strong as the ambient magnetic field in Kansas City (45 μT) and were well above intensity alterations known to cause birds to ignore geomagnetic cues (Wiltschko, 1972). The other non-baseline conditions in Sastre et al. (2002), simulated conditions at the North and South poles.

Additionally, the EEG analytical techniques in common use have undergone a number of changes over the years. Time-frequency analysis using wavelet methods are now standard in most analysis packages and allow the analyst to examine time-varying power fluctuations across a range of latencies. In contrast, the direct application of Fourier transforms to EEG data provides average power levels within large pre-defined epochs. To test the impact of these differences in data analysis algorithms, we analyzed our data using the techniques in Sastre et al. (2002). These analyses did not reveal any significant differences in total or band-specific power between any of our conditions. This suggests that, if neural responses were present in the Sastre et al. (2002) study, they may not have been revealed by the analyses used at the time.

Recent studies have also revealed that radio-frequency (Rf) noise can cause confounds in magnetoreception studies. Exposure to Rf noise has been shown to shut down magnetoreceptivity in birds and other animals (Engels et al., 2014; Landler et al., 2015; Wiltschko et al., 2015; Tomanova and Vacha, 2016). This is theorized to allow animals to cope with natural events such as solar storms, which cause the magnetic field to become unreliable as a navigational cue. Equivalent levels of Rf noise are also frequently present in our modern environment. Thus, experiments conducted in unshielded conditions may yield negative or fluctuating results due to uncontrolled Rf exposures.

Finally, there is a conceptual distinction to be made between studies examining potential health risks associated with electromagnetic fields and our present study looking for neural transduction. The former looks for physically-driven impacts of (usually high-energy) fields, whereas we look for biologically-driven responses to ambient-strength fields. High-energy fields can of course induce currents in, or even cause damage to nervous tissue. However, what we find in our study is indicative of a biological mechanism in action due to its selective response among energetically equivalent stimuli. The results suggest a neural response that has been tuned by natural selection to distinguish between ecologically-relevant magnetic field stimuli, versus other stimuli which would not be found naturally in the local environment.

Future experiments should examine how magnetoreceptive processing interacts with other sensory modalities to determine field orientation. Our experimental results suggest the combination of a magnetic and a positional cue (e.g., reacting differently to North-up and North-down fields). However, we cannot tell if this positional cue uses a reference frame set by gravity sensation (as in birds) or is aligned with respect to the human body. The neural processing of magnetic with gravitational sensory cues could perhaps be addressed by modifying the test chamber to allow the participant to rest in different orientations with respect to gravity or by running experiments in a zero-gravity environment.

Other multimodal interactions of interest may also occur with the vestibular sensation, given its role in sensing bodily orientation and rotation. In the experiments presented here, the participants would have had strong vestibular cues that they were level and stationary. This may have suppressed conflicting magnetic cues or given rise to error signals. Future experiments could manipulate vestibular inputs to test for interactions with magnetic field responses, which could help us interpret what those responses encode.

Future studies should also examine individual differences in transduction responsiveness. In the participant pool, we found several highly responsive individuals whose alpha-ERD proved to be stable across time: 4 participants responded strongly at the *p* < 0.01 level in repeated testing over weeks or months. Repeatability in those participants suggests that the alpha-ERD did not arise due to chance fluctuations in a single run but instead reflects a consistent individual characteristic, measurable across multiple runs. A wider survey of individuals could reveal genetic/developmental or other systematic differences underlying these individual differences.

The range of individual responses may be partially attributed to variation in basic alpha-ERD mechanisms rather than to underlying magnetoreceptive processing. However, some participants with high resting alpha-power showed very little alpha-ERD to the magnetic field rotations, suggesting that the extent of magnetoreceptive processing itself varies across individuals. If so, distinct human populations may be good targets for future investigation. For example, studies of comparative linguistics have identified a surprising number of human languages that rely on a cardinal system of environmental reference cues (e.g., North, South, East, West) and lack egocentric terms like front, back, left, and right (Haviland, 1998; Levinson, 2003; Meakins, 2011; Meakins and Algy, 2016; Meakins et al., 2016). Native speakers of such languages would, e.g., refer to a nearby tree as being to their North rather than being in front of them; they would refer to their own body parts in the same way. Individuals who have been raised from an early age within a linguistic, social and spatial framework using cardinal reference cues might have made associative links with geomagnetic sensory cues to aid in daily life; indeed, linguists have suggested a human magnetic compass might be involved (Levinson, 2003). It would be interesting to test such individuals using our newly-developed methods to see whether such geomagnetic cues might already be more strongly encoded, aiding their use of the cardinal reference system.

In the 199 years since Danish physicist Hans Christian Ørsted discovered electromagnetism (March 1820), human technology has made ever-increasing use of it. Most humans no longer need to rely on an internal navigational sense for survival. To the extent that we employ a sense of absolute heading in our daily lives, external cues such as landmarks and street grids can provide guidance. Even if an individual possesses an implicit magnetoreceptive response, it is likely to be confounded by disuse and interference from our modern environment. A particularly pointed example is the use of strong permanent magnets in both consumer and aviation headsets, most of which produce static fields through the head several times stronger than the ambient geomagnetic field. If there is a functional significance to the magnetoreceptive response, it would have the most influence in situations where other cues are impoverished, such as marine and aerial navigation, where spatial disorientation is a surprisingly persistent event (Poisson and Miller, 2014). The current alpha-ERD evidence provides a starting point to explore functional aspects of magnetoreception by employing various behavioral tasks in a variety of sensory settings.

## Conclusion

Our results indicate that at least some modern humans transduce changes in Earth-strength magnetic fields into an active neural response. We hope that this study provides a road-map for future studies aiming to replicate and extend research into human magnetoreception. Given the known presence of highly-evolved geomagnetic navigation systems in species across the animal kingdom, it is perhaps not surprising that we might retain at least some functioning neural components especially given the nomadic hunter/gatherer lifestyle of our not-too-distant ancestors. The full extent of this inheritance remains to be discovered.

## Synthesis

Reviewing Editor: Leonard Maler, University of Ottawa Decisions are customarily a result of the Reviewing Editor and the peer reviewers coming together and discussing their recommendations until a consensus is reached. When revisions are invited, a fact-based synthesis statement explaining their decision and outlining what is needed to prepare a revision will be listed below. The following reviewer(s) agreed to reveal their identity: Michael Winklhofer, Eric Warrant.

Editorial comments.

The authors are to be congratulated. Both reviewers and the editor agreed that this was a very fine piece of work; a great deal of attention was paid to the controls and the results therefore set a new standard in this field. This paper will be highly cited and its implications will resonate with both neuroscientists and the public.

Both reviewers did find some points that need attention from the authors. However, the revisions will not require re-review but only a check from the editor. Please make sure that you have an explicit 'response to comments' and a redlined version of your revision.

I will briefly summarize the main points re revisions agreed upon by the reviewers. And I also include all the careful detailed comments because they will prove helpful in your revision of the Ms.

The CW and CCW response asymmetry means that the EEG responses are unlikely to have been caused by induction artifacts. It might be very interesting to see whether lateralization is important when discussing the CW-CCW asymmetry.

Head turns to the right may be important for sensing the magnetic field (and whether this is connected to being predominantly left or right handed) is potentially an important insight and an an easy follow up with their subjects. 

The authors should show a band-pass filtered trace (in the time domain), as well as a typical induction-related artifact for the CW and CCW stimuli (again in the time domain). This would be helpful for readers unfamiliar with the EEG technique.

It would be very helpful to use colour maps in Matlab to increase the visual impact of their data. 

Reviewer #1

A range of vertebrates have a magnetic sense that allows them to use orientation cues from the Earth's magnetic field. Although modern humans are not known to consciously perceive the magnetic field, they still may have vestigial structures that mediate some subconscious responses to 

earth-strength magnetic fields (MFs). An earlier EEG study by Sastre et al. 2002 found no conclusive evidence of such responses, that is, the only statistically notable effects were observed under “North Pole” conditions in the low-frequency bands (Delta/Theta), where spectral power was higher compared to baseline exposure. Those effects were considered spurious, obviously because the variability in spectral power was generally larger across test conditions (changes in field geometry) than across exposure conditions (test vs sham). 

The authors of the present manuscript made a great effort to implement highly controlled electromagnetic test conditions in their test chambers where they performed EEG recordings from a total of 34 volunteers, in a fully blinded manner. They report an intriguing effect of MF changes on alpha-oscillations, which drop in power when the declination (i.e. horizontal magnetic field component) shifts CCW, but not CW (and when inclination is pointing down, as on Northern hemisphere). The observed effect is most pronounced anterior and indistinguishable from a well-known EEG pattern, the so-called event-related alpha desynchronization, which typically occurs when neurons switch from rhythmical activity to processing mode in response to incoming (sensory) information. The spectral evolution diagrams show that the effect does not appear immediately after stimulation and is of relatively short duration (0.2 s), so that it would go unnoticed if one were to apply just the overall Fourier spectrum analysis approach by Sastre et al. 2002. 

The CCW response is evident (e.g., Fig. 4, SI movies), reproducible (Fig. 4C, 7), and statistically sound (Fig. 5b), although it is a riddle why a CW shift does not elicit similar neural responses. On the other hand, that asymmetry means that the EEG responses are unlikely to have been caused by induction artefacts. 

To constrain the mechanism by which the magnetic field is received in primary sensory nerves, the authors performed several tests on the basis of the symmetry arguments depicted in Fig.3. A radical-pair based magnetoreception mechanism (quantum compass) would be intrinsically axial and should show antipodal symmetry while induction would give an undistinguishable response for identical changes in the B vector (Eq. 1 & 2). Both tests were negative (i.e. null hypothesis of undistinguishable responses could not be refuted), so that the authors single out the magnetite mechanism. Magnetite-based torque receptors, characterized by the cross product m x B, defy such symmetry tests because it is not generally possible to find a second B-vector that gives the same cross product m x B for N arbitrarily oriented m vectors in a population of N sensory cells. An alternative torque detector that merely measures the magnitude of the torque behaves axial (Winklhofer & Kirschvink 2010), can be ruled out on the basis of the negative axiality test (“quantum compass”). Yet another alternative magnetite detector that measures the rate of change in torque, m x dB/dt, can be ruled out on grounds of the negative induction test (identical dB/dt change applied, but from different B vector orientations, which yields different torque vectors despite identical rate of change in torque). 

That leaves a proper torque receptor, sensitive to the total torque vector. 

The linguistic aspects in the general discussion of the paper are of course speculative at this stage, but very interesting nevertheless. A paragraph about lateralization would be an interesting addition when discussing the CW-CCW asymmetry. In the head centered frame, a right turn (cw) of the head is equivalent to a ccw shift of the magnetic field and vice versa, which could suggest that head turns to the right are important for sensing the magnetic field. Kinsbourne (1972, Science) observed head turns when test persons had to solve cognitive problems and found that the head turn direction depends on the type of problem (verbal vs spatial), with inverse results between left-handed and right-handed people. With that in mind, I'd encourage the authors to find out if the persons who responded to CCW field shifts were predominantly left handed or right handed. 

In sum, the paper presents exciting results - and the first evidence of human magnetoreception, obtained under meticulously controlled conditions. The authors have minimized all known artefacts and describe in great detail the experimental setup and procedures so that anyone can in principle engage in a replication study. I therefore strongly recommend publication after some of the minor points below have been taken care of: 

1) and most importantly: the authors should show a band-pass filtered trace (i.e. in the time domain), (e.g. for electrode Fz), so as to give readers an idea of how strongly the alpha band “flattens” out in response to the DecDn.CCW.N stimulus because a 1 dB reduction in power (Fig 5.b), does not appear a large effect to EEG outsiders, although it is a typical reduction observed in EEG alpha ERD (1 db is 25%). 

Along these lines, the authors should also show typical induction-related artefacts for both CW and CCW stimulus, also in the time domain. 

2) Fig. 5A-C please do not show bar diagrams but either boxplots or points with mean and S.D. 

3) Repeated-measurement AOV results (Table 1). Is eta_p^2 the effect size after Friedman 1968? 

4) Fig. 4 A,B The flashy color scheme used (matlab 'jet' ?) is o.k. but not ideal because the color ordering is not perceptual, see 

http://medvis.org/2012/08/21/rainbow-colormaps-what-are-they-good-for-absolutely-nothing/ 

For that reason, matlab has replaced jet by parula in the default color map. 

5) When discussing the putative transduction mechanism, it would be useful to show the torque equation m x b, perhaps with a sketch of the magnetite mechanism. 

6) References: 

Regarding design for biomagnetic experiments, these two reference are useful too 

Schwarze et al. 2016 ( 10.3389/fnbeh.2016.00055 ) 

Kirschvink et al. 2010 ( 10.1098/rsif.2009.0491.focus ). 

7) When discussing why Sastre et al. 2002 may have missed MF effects (p. 27), the authors of the present study offer have several explanations, of which the usage of a spectral analysis technique without temporal resolution is the most plausible one (although the matlab wavelet analysis program by Torrence and Compo (1998, Bull. Amer. Meteor. Soc.) would have been available then ... ) . 

The other possible explanation invoking functional window of magnetic field parameters is more speculative. The conditions in the Sastre et al. study were not so grossly unnormal compared to the three times earth-field MF strength that Walker et al. 1997 (Nature) applied to identify MF-responsive neurons in trout (mediated by a putative magnetite receptor). In birds, Heyers et 2010 (PNAS) found strong neuronal activation in trigeminal brain-stem nuclei when the ambient field strength changed from half to twice its ambient intensity value. That would imply that unnormal fields are rejected at a higher integration center if they are rejected at all. So far the only evidence for a functional window applies to inclination compass responses mediated by putative radical-pair molecules. 

Reviewer #2

In this rather remarkable study, the authors perform carefully controlled experiments to investigate the brainwave activity of human subjects in response to specific rotations of the inclination and declination of an Earth-strength magnetic field. The amplitude of this brainwave activity - EEG alpha oscillations between 8 and 13 Hz, termed alpha event-related desynchronization (alpha-ERD) - declined significantly in some (but interestingly not all) human participants as a result of horizontal rotations of the magnetic field when the static vertical component of the field was directed downwards (as in the northern hemisphere), but not when it was directed upwards. Moreover, alpha-ERD amplitude also declined during counterclockwise declination rotations (with downward inclination). However, during clockwise rotations, or with upward inclination, alpha-ERD amplitude remained unchanged. Since declines in alpha-ERD amplitude have previously been measured in human responses to stimulation from other sensory modalities, the authors conclude that the changes in alpha-ERD amplitude which they measured in response to specific changes in an Earth-strength magnetic field imply that some humans therefore have the ability to both sense and transduce this field. Interestingly this ability comes without a conscious perception of the magnetic field, a finding that the authors suggest could reveal remnants of an ancestral magnetic sense that we no longer use. 

The authors were further able to narrow down the possible sensory mechanism employed by humans to detect the earth's magnetic field. They show that changes in alpha-ERD amplitude were dependent on the polarity of the field, implying that the quantum hypothesis of magnetic reception cannot explain these results. Further experiments showed that the neural response was sensitive to static components of the field, revealing that electrical induction (which depends on dynamic components) likewise cannot explain the results. Thus, the authors conclude that a mechanism based on biogenic magnetite remains the only mechanism of magnetic reception that could subserve a human magnetic sense. 

Normally such results would be considered highly controversial, especially in light of the rather fractious history of studies investigating a purported magnetic sense in humans. However, I was impressed by the rigour and care with which this study was performed. Enormous efforts have been made to eliminate obvious potential sources of artifact (and almost a third of the manuscript is devoted to explaining how they have been eliminated). The double-wound coil system allowed sham control experiments to be performed, the experimental room was heavily shielded from external sources of RF noise, all potentially noisy equipment was placed 20 m from the apparatus, and only non-magnetic materials were permitted in the experimental zone. Moreover, precise measurements of the field were continuously made at the centre of the coils, and negligible levels of RF noise were also measured with state-of-the-art equipment. It is very apparent that the experimenters knew exactly what they were doing, which unfortunately is not always the case in this field. I thus trust the quality of the stimulation they have provided. The results that they have obtained using these stimuli also seem to be sound, although I do confess I am unfamiliar with EEG techniques and am unable to judge the quality or validity of the measurements they have made using these methods. However, assuming that the alpha-ERD amplitudes have been correctly measured, then my conclusion is that the results of the study are highly significant and of major interest to the scientific community. Obviously, these results stimulate more questions than they answer, and I am sure this study will stimulate a new wave of research on this curious (but likely unused) human sense. 

The manuscript, while lengthy, is beautifully written and illustrated. I found only one typing error and the nature of my (very few) comments below are more generated by curiosity than criticism. 

1. Line 512. You show that only some individuals neurally respond to changes in the magnetic field, and that these have a high resting-state alpha power. Is this a permanent state present in some individuals, or is this something that varies from one day to the next, or from one time of the year to another, all from one emotional state to another? In other words, is it possible that all humans could show a response if in the right “frame of mind”? 

2. Following on from this question, what is the situation for measurements of alpha-ERD amplitude during stimulation in other sensory modalities? Is it also the case that only some individuals show a response while others don't? Or is it the case that all individuals show a strong response and that this is because vision or hearing (say) are much more important senses for humans? It may be the case that the more hit-and-miss responses you have measured during magnetic stimulation may simply be due to the lack of importance of a magnetic sense in humans. This might be worth taking up in the Discussion. 

3. Line 712. Insert “at” after “arise”. 

4. Line 806: “previous” not “Previous” 

5. Lines 870-876: These observations about tribes of native people describing the locations of objects relative to environmental reference cues is interesting but there are potentially other cues available other than the earth's magnetic field for distinguishing such cues. These would include celestial cues such as the sun during the day or stars at night. 

6. Fig. 4C. I am unable to see the pink vertical lines referred to in this figure, and even the white outlines mentioned are difficult to see. Could these be improved? 

## Author Response

Synthesis of Reviews:

Significance Statement Comments for Author (Required):

OK

Comments on the Visual Abstract for Author (Required):

N/A

Synthesis Statement for Author (Required):

Editorial comments.

The authors are to be congratulated. Both reviewers and the editor agreed that this was a very fine piece of work; a great deal of attention was paid to the controls and the results therefore set a new standard in this field. This paper will be highly cited and its implications will resonate with both neuroscientists and the public.

Thank you.

Both reviewers did find some points that need attention from the authors. However, the revisions will not require re-review but only a check from the editor. Please make sure that you have an explicit 'response to comments' and a redlined version of your revision.

Our 'responses to comments' are in this blue font following each comment, and the

'redlined' revisions are recorded in 'Track Changes' mode on the attached MS Word version of the document (Wang_etal_REVISED_tracked.docx)

I will briefly summarize the main points re revisions agreed upon by the reviewers. And I also include all the careful detailed comments because they will prove helpful in your revision of the Ms.

The CW and CCW response asymmetry means that the EEG responses are unlikely to have been caused by induction artifacts. It might be very interesting to see whether lateralization is important when discussing the CW-CCW asymmetry.

We agree, but as of yet this does not seem to be related to the Left-handed vs. Right-handed information provided to us by the Participants in our experiment. This CW vs. CCW asymmetry appears strongly in the current experimental paradigm, but we would not be surprised if further research reveals occasional subjects who respond to both, or the opposite, pattern. Certainly, subjects living on the geomagnetic equator where the field is horizontal, or in the Southern Hemisphere where the North-seeking magnetic vector points upwards might be expected to have different response patterns. That is one of the promising future directions, beyond the scope of our present study.

Head turns to the right may be important for sensing the magnetic field (and whether this is connected to being predominantly left or right handed) is potentially an important insight and an easy follow up with their subjects.

Although our subjects are sitting in the dark, we monitor them continuously with an IR video camera. We have never noticed subjects turning their heads - most simply sit back with their eyes closed. Head motions of that sort would also generate characteristic EEG patterns in the most distal electrodes that are closest to muscles controlling the head orientation, and those would have shown up as a separate (non alpha-ERD) response when time-locked with the magnetic signature.

These do not appear in our data. Having said this, however, it might still be interesting to see if the participant can improve its discrimination performance, by turning its head (or entire trunk). Again, that is beyond the scope of this study.

The authors should show a band-pass filtered trace (in the time domain), as well as a typical induction-related artifact for the CW and CCW stimuli (again in the time domain). This would be helpful for readers unfamiliar with the EEG technique.

We agree. We have added a figure to the MS (New Figure 4) that contains time-domain single trial traces of a typical individual's EEG response to the inclination and declination sweep experiments, as well as a comparison with a standard EEG phantom (a cantaloupe) which allows the reader to clearly see the difference between the brief electrical artifacts, and the typical alphawave pattern for brains with weak and strong alpha-wave patterns.

The description of this has been added to the text in lines 161-168, as follows:

“Examples of single-trial, time-domain, bandpass-filtered (1-50 Hz) EEG traces at electrode Fz are shown in Figure 4. Figure 4A shows the artifact during the inclination rotation, measured from a cantaloupe and a human. The artifact is detectable in single trials from participants with low alpha power (as shown), but difficult to see in participants with high alpha power. Figure 4B shows the induction artifact during the declination rotation, which has smaller ¶B/¶t and produces a smaller artifact. The artifact is visible in the cantaloupe trace, but typically invisible in single-trial human

EEG.”

Also in the Results section on lines 541-544 we add:

“Figure 4B shows a sample EEG voltage trace that contains such a drop in alpha power. The time-frequency power maps in Figure 5 are cross-trial averages and show how the spectral power contained in the EEG trace changed across time. Drops in power are depicted in a deep blue color. ”

It would be very helpful to use colour maps in Matlab to increase the visual impact of their data.

We agree, again. Rather than using the rainbow-colored default map in the Matlab generated plots in our current figures, we have changed the plots and movies to use the colorblindfriendly blue-yellow map, parula. This has necessitated a few changes of color terminology throughout the text, which have been 'tracked', as well as changing the db scale from +/-4 to +/-3 on the color maps.

Reviewer #1

A range of vertebrates have a magnetic sense that allows them to use orientation cues from the Earth's magnetic field. Although modern humans are not known to consciously perceive the magnetic field, they still may have vestigial structures that mediate some subconscious responses to earth-strength magnetic fields (MFs). An earlier EEG study by Sastre et al. 2002 found no conclusive evidence of such responses, that is, the only statistically notable effects were observed under “North Pole” conditions in the low-frequency bands (Delta/Theta), where spectral power was higher compared to baseline exposure. Those effects were considered spurious, obviously because the variability in spectral power was generally larger across test conditions (changes in field geometry) than across exposure conditions (test vs sham).

Agreed.

The authors of the present manuscript made a great effort to implement highly controlled electromagnetic test conditions in their test chambers where they performed EEG recordings from a total of 34 volunteers, in a fully blinded manner. They report an intriguing effect of MF changes on alpha-oscillations, which drop in power when the declination (i.e. horizontal magnetic field component) shifts CCW, but not CW (and when inclination is pointing down, as on Northern hemisphere). The observed effect is most pronounced anterior and indistinguishable from a wellknown EEG pattern, the so-called event-related alpha desynchronization, which typically occurs when neurons switch from rhythmical activity to processing mode in response to incoming (sensory) information. The spectral evolution diagrams show that the effect does not appear immediately after stimulation and is of relatively short duration (0.2 s), so that it would go unnoticed if one were to apply just the overall Fourier spectrum analysis approach by Sastre et al.

2002.

Agreed.

The CCW response is evident (e.g., Fig. 4, SI movies), reproducible (Fig. 4C, 7), and statistically sound (Fig. 5b), although it is a riddle why a CW shift does not elicit similar neural responses. On the other hand, that asymmetry means that the EEG responses are unlikely to have been caused by induction artefacts.

Agreed on both counts. We think it is more likely that this asymmetry arises at the level of neural processing, rather than at the biophysical transduction step. Hence, we might find other magnetically-influenced patterns that do not have such asymmetry. We are working on the next step to establish a sensitive, implicit behavioral measure like reaction time for detection of subtle magnetoreceptive discrimination, and then to see if there is qualitatively the same asymmetry at such an implicit behavioral level in the majority of “responders.” However, that is beyond the scope of this present manuscript.

To constrain the mechanism by which the magnetic field is received in primary sensory nerves, the authors performed several tests on the basis of the symmetry arguments depicted in Fig.3. A radical-pair based magnetoreception mechanism (quantum compass) would be intrinsically axial and should show antipodal symmetry while induction would give an undistinguishable response for identical changes in the B vector (Eq. 1 & 2). Both tests were negative (i.e. null hypothesis of undistinguishable responses could not be refuted), so that the authors single out the magnetite mechanism. Magnetite-based torque receptors, characterized by the cross product m x B, defy such symmetry tests because it is not generally possible to find a second B-vector that gives the same cross product m x B for N arbitrarily oriented m vectors in a population of N sensory cells. An alternative torque detector that merely measures the magnitude of the torque behaves axial (Winklhofer & Kirschvink 2010), can be ruled out on the basis of the negative axiality test (“quantum compass”). Yet another alternative magnetite detector that measures the rate of change in torque, m x dB/dt, can be ruled out on grounds of the negative induction test (identical dB/dt change applied, but from different B vector orientations, which yields different torque vectors despite identical rate of change in torque).

That leaves a proper torque receptor, sensitive to the total torque vector. Agreed, as we conclude for similar reasons.

The linguistic aspects in the general discussion of the paper are of course speculative at this stage, but very interesting nevertheless. A paragraph about lateralization would be an interesting addition when discussing the CW-CCW asymmetry. In the head centered frame, a right turn (cw) of the head is equivalent to a ccw shift of the magnetic field and vice versa, which could suggest that head turns to the right are important for sensing the magnetic field. Kinsbourne (1972, Science) observed head turns when test persons had to solve cognitive problems and found that the head turn direction depends on the type of problem (verbal vs spatial), with inverse results between left-handed and right-handed people. With that in mind, I'd encourage the authors to find out if the persons who responded to CCW field shifts were predominantly left handed or right handed.

Of our four strongest subjects, 3 are right-handed, and 1 is left-handed, and all are CCW responders. Because only about 10% of humans identify as left-handed, it would take a much larger sample size to test for an association of this sort, and that is beyond the scope of this present manuscript.

In sum, the paper presents exciting results - and the first evidence of human magnetoreception, obtained under meticulously controlled conditions. The authors have minimized all known artefacts and describe in great detail the experimental setup and procedures so that anyone can in principle engage in a replication study. I therefore strongly recommend publication after some of the minor points below have been taken care of:

1) and most importantly: the authors should show a band-pass filtered trace (i.e. in the time domain), (e.g. for electrode Fz), so as to give readers an idea of how strongly the alpha band “flattens” out in response to the DecDn.CCW.N stimulus because a 1 dB reduction in power (Fig 5.b), does not appear a large effect to EEG outsiders, although it is a typical reduction observed in EEG alpha ERD (1 db is 25%).

Thank you - this is a good idea. The new Figure 4 mentioned above shows example traces from both low and high-alpha human brains, for inclination and declination sweeps, and compares them with matched data from a phantom (the cantaloupe) where the induction artifact can be recognized clearly. Note that adding this figure has caused the figures that follow to be renumbered.

Along these lines, the authors should also show typical induction-related artefacts for both CW and CCW stimulus, also in the time domain.

Again, this is now on the new Figure 4, and is clearly seen on the cantaloupe data.

2) Fig. 5A-C please do not show bar diagrams but either boxplots or points with mean and S.D.

The individual participant data are shown on the histograms of Fig. 7 (previous Figure 6), so all of the data are actually there. The bar diagrams, with the histograms, are in standard use in the field, so we prefer to keep them.

3) Repeated-measurement AOV results (Table 1). Is eta_p^2 the effect size after Friedman 1968?

No - it is the partial eta-squared value. To clarify this we have added this sentence to the end of the caption for Tables 1-3: “On this table, F is the F-ratio statistic, p the probability value, and ηp2 the partial eta-squared value from the ANOVA.”

4) Fig. 4 A,B The flashy color scheme used (matlab 'jet' ?) is o.k. but not ideal because the color ordering is not perceptual, see

http://medvis.org/2012/08/21/rainbow-colormaps-what-are-they-good-for-absolutely-nothing/ ; For that reason, matlab has replaced jet by parula in the default color map.

All of our images, and the movies, now use the standard parula (bue-yellow) color map. This has caused some minor changes in the text to reflect the new color standards. We have even altered Figure 3 so as to change the green arrows to blue, thereby avoiding red/green comparison. Additionally, we have tested our figures vis-à-vis a color-blind colleague to ensure that they are easy to view.

5) When discussing the putative transduction mechanism, it would be useful to show the torque equation m x b, perhaps with a sketch of the magnetite mechanism.

This torque relationship is mentioned in the paragraph, “Controlling for

Magnetomechanical Artifacts”, lines 472-492 of the revised text. Rather than adding an additional figure we refer simply to the discussions in Kirschvink (1992a) and Winklhofer & Kirschvink (2010). In the General Discussion, we have added the following text:

“In the simplest form, the torque (= u x B) from a string of magnetite crystals (a 'magnetosome chain' like those in the magnetotactic bacteria) could act to open and close trans-membrane ion channels. Several biophysical analyses have shown this is a most plausible mechanism (Kirschvink, 1992a; Winklhofer and Kirschvink, 2010).”, This is now in lines 808-810 of the revised ms.

6) References:

Regarding design for biomagnetic experiments, these two reference are useful too Schwarze et al. 2016 ( 10.3389/fnbeh.2016.00055 ) Kirschvink et al. 2010 ( 10.1098/rsif.2009.0491.focus ).

Agreed. We have added to the caption of Fig. 2: “Additional guidance for the design of biomagnetic experiments is given by Kirschvink et al. (2010) and Schwarze et al. (2016)”, lines 1262-1263 of the revised manuscript.

7) When discussing why Sastre et al. 2002 may have missed MF effects (p. 27), the authors of the present study offer have several explanations, of which the usage of a spectral analysis technique without temporal resolution is the most plausible one (although the matlab wavelet analysis program by Torrence and Compo (1998, Bull. Amer. Meteor. Soc.) would have been available then

... ) .

True, but the Torrence and Compo method was not used by Sastre et al. Furthermore, we note that our data analyzed by the techniques of Sastre et al. did not reveal evidence for magnetoreception; this is mentioned in lines 867-871. We did contact Sastre to see if their data from the late 1990s were available to run through our analyses, but it seems that the magnetic tapes containing their raw data were recycled or discarded many years ago.

On line 55 of the Introduction, we have therefore changed “... were limited by computational methods of the time (Sastre et al., 2002)” with “... were limited by the computations methods that were used (Sastre et al., 2002).”

The other possible explanation invoking functional window of magnetic field parameters is more speculative. The conditions in the Sastre et al. study were not so grossly unnormal compared to the three times earth-field MF strength that Walker et al. 1997 (Nature) applied to identify MFresponsive neurons in trout (mediated by a putative magnetite receptor). In birds, Heyers et 2010 (PNAS) found strong neuronal activation in trigeminal brain-stem nuclei when the ambient field strength changed from half to twice its ambient intensity value. That would imply that unnormal fields are rejected at a higher integration center if they are rejected at all. So far the only evidence for a functional window applies to inclination compass responses mediated by putative radical-pair molecules.

We certainly agree with this statement. The magnetoreceptor cells are most likely doing their best to transduce the vector direction and perhaps intensity of the magnetic field as they experience it. In both the Walker et al. (1997) and Heyers et al. (2010) studies the authors were able show that those intensity fluctuations were making it into the animals' nervous system. And

Walker's fish certainly were able to be conditioned in a simple training paradigm to field fluctuations similar to those used in the electrophysiological recordings, implying that the information was not being filtered out by the brain prior to the elicitation of a behavioral response in fish as it is in birds. Although we do not report the results of intensity fluctuations on humans in this study, we did find strong evidence for neural filtering of the inclination (e.g., upwards directed fields elicit no alpha-ERD). The whole point here was to illustrate that there are other examples of such neural filtering of magnetic information; hence, we would rather not modify the discussion on this point.

Reviewer #2

In this rather remarkable study, the authors perform carefully controlled experiments to investigate the brainwave activity of human subjects in response to specific rotations of the inclination and declination of an Earth-strength magnetic field. The amplitude of this brainwave activity - EEG alpha oscillations between 8 and 13 Hz, termed alpha event-related desynchronization (alpha-ERD)

- declined significantly in some (but interestingly not all) human participants as a result of horizontal rotations of the magnetic field when the static vertical component of the field was directed downwards (as in the northern hemisphere), but not when it was directed upwards. Moreover, alpha-ERD amplitude also declined during counterclockwise declination rotations (with downward inclination). However, during clockwise rotations, or with upward inclination, alphaERD amplitude remained unchanged. Since declines in alpha-ERD amplitude have previously been measured in human responses to stimulation from other sensory modalities, the authors conclude that the changes in alpha-ERD amplitude which they measured in response to specific changes in an Earth-strength magnetic field imply that some humans therefore have the ability to both sense and transduce this field. Interestingly this ability comes without a conscious perception of the magnetic field, a finding that the authors suggest could reveal remnants of an ancestral magnetic sense that we no longer use.

We agree with this summary.

The authors were further able to narrow down the possible sensory mechanism employed by humans to detect the earth's magnetic field. They show that changes in alpha-ERD amplitude were dependent on the polarity of the field, implying that the quantum hypothesis of magnetic reception cannot explain these results. Further experiments showed that the neural response was sensitive to static components of the field, revealing that electrical induction (which depends on dynamic components) likewise cannot explain the results. Thus, the authors conclude that a mechanism based on biogenic magnetite remains the only mechanism of magnetic reception that could subserve a human magnetic sense.

Again, we agree with this summary.

Normally such results would be considered highly controversial, especially in light of the rather fractious history of studies investigating a purported magnetic sense in humans. However, I was impressed by the rigour and care with which this study was performed. Enormous efforts have been made to eliminate obvious potential sources of artifact (and almost a third of the manuscript is devoted to explaining how they have been eliminated). The double-wound coil system allowed sham control experiments to be performed, the experimental room was heavily shielded from external sources of RF noise, all potentially noisy equipment was placed 20 m from the apparatus, and only non-magnetic materials were permitted in the experimental zone. Moreover, precise measurements of the field were continuously made at the centre of the coils, and negligible levels of RF noise were also measured with state-of-the-art equipment. It is very apparent that the experimenters knew exactly what they were doing, which unfortunately is not always the case in this field. I thus trust the quality of the stimulation they have provided. The results that they have obtained using these stimuli also seem to be sound, although I do confess I am unfamiliar with EEG techniques and am unable to judge the quality or validity of the measurements they have made using these methods. However, assuming that the alpha-ERD amplitudes have been correctly measured, then my conclusion is that the results of the study are highly significant and of major interest to the scientific community. Obviously, these results stimulate more questions than they answer, and I am sure this study will stimulate a new wave of research on this curious (but likely unused) human sense.

We agree.

The manuscript, while lengthy, is beautifully written and illustrated. I found only one typing error and the nature of my (very few) comments below are more generated by curiosity than criticism.

1. Line 512. You show that only some individuals neurally respond to changes in the magnetic field, and that these have a high resting-state alpha power. Is this a permanent state present in some individuals, or is this something that varies from one day to the next, or from one time of the year to another, all from one emotional state to another? In other words, is it possible that all humans could show a response if in the right “frame of mind”?

As described with the example in Figure 5 (formerly Fig. 4), many of our participants display a strong and persistent pattern in their alpha-ERD response that lasts stably for weeks to months, and even years, and they can yield highly significant results individually. These participants are usually characterized by strong power in their alpha band, as shown from the human example in the new Fig. 4B. We have found, however, that an occasional individual with otherwise strong alpha power does not display a magnetic response, so strong alpha power alone does not imply that an individual has a good magnetoreceptive ability. Other individuals, however, have alpha-waves that are intrinsically weaker and may be highly variable (e.g., the human in Fig. 4A). Whereas alpha wave power itself is modulated by internal state (such as drowsiness, or alert/attention level), there are clear individual differences beyond it, thus it is considered to be one of the dynamic “fingerprints” of each individual brain. Our test-retest results show this stability, both in terms of alpha peak frequency, power, and the degree of its suppression

(desynchronization) in response to magnetic field rotations. If an individual or a group of individuals have either low baseline alpha band power (low signal), or highly variable baseline alpha band power (high noise), then it makes it statistically difficult to distinguish magneticallytriggered alpha-ERD responses from background fluctuations in alpha-power. That said, fluctuations in alpha band power may relate to the level of distractedness or tiredness of the participant: i.e. the participant's “frame of mind”. If the participant is both more relaxed (less background distraction) and more alert, then the signal to noise ratio in the alpha-band power may improve. This suggests possible new experimental protocols that incorporate behavioral components to better focus our participant's attention during each experimental trial. These, however, are follow-on studies beyond the scope of the present manuscript.

2. Following on from this question, what is the situation for measurements of alpha-ERD amplitude during stimulation in other sensory modalities? Is it also the case that only some individuals show a response while others don't? Or is it the case that all individuals show a strong response and that this is because vision or hearing (say) are much more important senses for humans? It may be the case that the more hit-and-miss responses you have measured during magnetic stimulation may simply be due to the lack of importance of a magnetic sense in humans. This might be worth taking up in the Discussion.

In other modalities such as auditory, visual, and tactile, the strength of alpha-ERD is usually modulated by peak amplitude of alpha, as well as task context, attention, etc., as we discuss in the text on lines 558-569. Not everyone has the same alpha-ERD magnitude. Individual magnitudes can vary from 0-90%. However, we think it is yet too premature to comment on the lack of importance in modern humans.

3. Line 712. Insert “at” after “arise”.

Done, now on line 756.

4. Line 806: “previous” not “Previous” Done, now on line 855.

5. Lines 870-876: These observations about tribes of native people describing the locations of objects relative to environmental reference cues is interesting but there are potentially other cues available other than the earth's magnetic field for distinguishing such cues. These would include celestial cues such as the sun during the day or stars at night.

It is certainly true that most animals seem to use geomagnetic cues as the 'last line of resort' when navigating or homing. The sun and star compasses in birds, for example, will be used instead of magnetic cues when they are put in conflict. Native Speakers of the human languages that are based on cardinal orientation systems will also certainly use sun and star cues, but as noted by several authors (e.g., Levinson and Meakins in particular) their use of this system extends to situations where other cues are absent (e.g., cloudy nights, indoors, etc.). This is why we mention these studies, as an innate magnetic orientation capability (even if subconscious) could explain these language peculiarities.

6. Fig. 4C. I am unable to see the pink vertical lines referred to in this figure, and even the white outlines mentioned are difficult to see. Could these be improved?

This has been fixed on the new color scheme; we are using thick vertical black lines for the stimulus interval, and pink and white lines for the p = 0.05 and p = 0.01 contours, uniformly on the renumbered Figures 5, 6 and 8.

## References

[B1] Able KP, Gergits WF (1985) Human navigation: attempts to replicate Baker’s displacement experiment In: Magnetite biomineralization and magnetoreception in organisms: a new biomagnetism (Kirschvink JL, Jones DS, MacFadden BJ, eds), pp 569–572. New York, NY: Plenum Press.

[B2] Baker RR (1980) Goal orientation by blindfolded humans after long-distance displacement: possible involvement of a magnetic sense. Science 210:555–557. 7423208 10.1126/science.7423208

[B3] Baker RR (1982) Human navigation and the 6th sense. New York, NY: Simon and Schuster.

[B4] Baker RR (1987) Human navigation and magnetoreception: the Manchester experiments do replicate. Animal Behav 35:691–704. 10.1016/S0003-3472(87)80105-7

[B5] Bazylinski DA, Schlezinger DR, Howes BH, Frankel RB, Epstein SS (2000) Occurrence and distribution of diverse populations of magnetic protists in a chemically stratified coastal salt pond. Chem Geol 169:319–328. 10.1016/S0009-2541(00)00211-4

[B6] Beason RC, Semm P (1996) Does the avian ophthalmic nerve carry magnetic navigational information? J Exp Biol 199:1241–1244. 9319100 10.1242/jeb.199.5.1241

[B7] Beason RC, Wiltschko R, Wiltschko W (1997) Pigeon homing: effects of magnetic pulses on initial orientation. Auk 114:405–415. 10.2307/4089242

[B8] Benjamini Y, Hochberg Y (1995) Controlling the false discovery rate: a practical and powerful approach to multiple testing. J R Stat Soc Series B Stat Methodol 57:289–300. 10.1111/j.2517-6161.1995.tb02031.x

[B9] Block SM (1992) Biophysical aspects of sensory transduction In: Sensory transduction (Corey DP, Roper SD, eds), p 424 Woods Hole, MA: Rockefeller University Press.

[B10] Boorman GA, Bernheim NJ, Galvin MJ, Newton SA, Parham FM, Portier CJ, Wolfe MS (1999) U.S. National Institute of Environmental Health Sciences (NIEHS). Report on health effects from exposure to power-line frequency electric and magnetic fields. Research Triangle Park, NC: National Institute of Environmental Health Sciences.

[B11] Carporzen L, Weiss BP, Gilder SA, Pommier A, Hart RJ (2012) Lightning remagnetization of the Vredefort impact crater: no evidence for impact-generated magnetic fields. J Geophys Res 117:E01007.

[B12] Cohen MX (2014) Analyzing neural time series data theory and practice preface. Cambridge, MA: MIT Press.

[B13] Counselman C (2013) Excellent, easy, cheap common-mode chokes. Natl Contest J Am Radio Relay League 41:3–5.

[B14] Diebel CE, Proksch R, Green CR, Neilson P, Walker MM (2000) Magnetite defines a vertebrate magnetoreceptor. Nature 406:299–302. 10.1038/35018561 10917530

[B15] Dunn JR, Fuller M, Zoeger J, Dobson J, Heller F, Hammann J, Caine E, Moskowitz BM (1995) Magnetic material in the human hippocampus. Brain Res Bull 36:149–153. 7895092 10.1016/0361-9230(94)00182-z

[B16] Eder SH, Cadiou H, Muhamad A, McNaughton PA, Kirschvink JL, Winklhofer M (2012) Magnetic characterization of isolated candidate vertebrate magnetoreceptor cells. Proc Natl Acad Sci USA 109:12022–12027. 10.1073/pnas.1205653109 22778440 PMC3409731

[B17] Elbers D, Bulte M, Bairlein F, Mouritsen H, Heyers D (2017) Magnetic activation in the brain of the migratory northern wheatear (*Oenanthe oenanthe*). J Comp Physiol A Neuroethol Sens Neural Behav Physiol 203:591–600. 10.1007/s00359-017-1167-7 28361169

[B18] Engels S, Schneider NL, Lefeldt N, Hein CM, Zapka M, Michalik A, Elbers D, Kittel A, Hore PJ, Mouritsen H (2014) Anthropogenic electromagnetic noise disrupts magnetic compass orientation in a migratory bird. Nature 509:353–356. 10.1038/nature13290 24805233

[B19] Ernst DA, Lohmann KJ (2016) Effect of magnetic pulses on Caribbean spiny lobsters: implications for magnetoreception. J Exp Biol 219:1827–1832. 10.1242/jeb.136036 27045095

[B20] Fillmore EP, Seifert MF (2015) Anatomy of the trigeminal nerve In: Nerves and nerve injuries (Tubbs RS, Rizk E, Shoja M, Loukas M, Barbaro N, Spinner R, eds), pp 319–350. San Diego, CA: Academic Press.

[B150] Fontanini A, Katz DB (2008) Behavioral states, network states, and sensory response variability. Journal of Neurophysiology 100:1160–1168. 10.1152/jn.90592.2008 18614753 PMC2544460

[B21] Frankel RB, Blakemore RP (1980) Navigational compass in magnetic bacteria. J Magn Magn Mater 15–18:1562–1564. 10.1016/0304-8853(80)90409-6

[B22] Gilder SA, Wack M, Kaub L, Roud SC, Petersen N, Heinsen H, Hillenbrand P, Milz S, Schmitz C (2018) Distribution of magnetic remanence carriers in the human brain. Sci Rep 8:11363. 10.1038/s41598-018-29766-z 30054530 PMC6063936

[B23] Gould JS, Able KP (1981) Human homing: an elusive phenomenon. Science 212:1061–1063. 7233200 10.1126/science.7233200

[B24] Hand E (2016) Polar explorer. Science 352:1508–1510. 10.1126/science.352.6293.1508 27339964

[B25] Hartmann T, Schlee W, Weisz N (2012) It’s only in your head: expectancy of aversive auditory stimulation modulates stimulus-induced auditory cortical alpha desynchronization. Neuroimage 60:170–178. 10.1016/j.neuroimage.2011.12.034 22209810

[B26] Haviland JB (1998) Guugu Yimithirr cardinal directions. Ethos 26:25–47. 10.1525/eth.1998.26.1.25

[B27] Holland RA (2010) Differential effects of magnetic pulses on the orientation of naturally migrating birds. J R Soc Interface 7:1617–1625. 10.1098/rsif.2010.0159 20453067 PMC2988258

[B28] Holland RA, Helm B (2013) A strong magnetic pulse affects the precision of departure direction of naturally migrating adult but not juvenile birds. J R Soc Interface 10:20121047. 10.1098/rsif.2012.1047 23389901 PMC3627120

[B29] Holland RA, Kirschvink JL, Doak TG, Wikelski M (2008) Bats use magnetite to detect the earth’s magnetic field. PLoS One 3:e1676. 10.1371/journal.pone.0001676 18301753 PMC2246016

[B30] Hore PJ, Mouritsen H (2016) The radical-pair mechanism of magnetoreception. Annu Rev Biophys 45:299–344. 27216936 10.1146/annurev-biophys-032116-094545

[B31] Irwin WP, Lohmann KJ (2005) Disruption of magnetic orientation in hatchling loggerhead sea turtles by pulsed magnetic fields. J Comp Physiol A Neuroethol Sens Neural Behav Physiol 191:475–480. 10.1007/s00359-005-0609-9 15765235

[B32] Johnsen S, Lohmann KJ (2008) Magnetoreception in animals. Phys Today 61:29–35. 10.1063/1.2897947

[B33] Kalmijn AJ (1981) Biophysics of geomagnetic-field detection. IEEE Trans Magn 17:1113–1124. 10.1109/TMAG.1981.1061156

[B34] Kirschvink J, Padmanabha S, Boyce C, Oglesby J (1997) Measurement of the threshold sensitivity of honeybees to weak, extremely low-frequency magnetic fields. J Exp Biol 200:1363–1368. 9319256 10.1242/jeb.200.9.1363

[B35] Kirschvink JL (1992a) Comment on “Constraints on biological effects of weak extremely-low-frequency electromagnetic fields.” Phys Rev A 46:2178–2184. 10.1103/PhysRevA.46.2178 9908363

[B36] Kirschvink JL (1992b) Uniform magnetic fields and double-wrapped coil systems: improved techniques for the design of bioelectromagnetic experiments. Bioelectromagnetics 13:401–411. 10.1002/bem.2250130507 1445421

[B37] Kirschvink JL, Gould JL (1981) Biogenic magnetite as a basis for magnetic field detection in animals. Biosystems 13:181–201. 7213948 10.1016/0303-2647(81)90060-5

[B38] Kirschvink JL, Kobayashi-Kirschvink A (1991) Is geomagnetic sensitivity real? Replication of the Walker-Bitterman conditioning experiment in honey bees. Am Zool 31:169–185. 10.1093/icb/31.1.169

[B39] Kirschvink JL, Kobayashi-Kirschvink A, Woodford BJ (1992) Magnetite biomineralization in the human brain. Proc Natl Acad Sci USA 89:7683–7687. 1502184 10.1073/pnas.89.16.7683 PMC49775

[B40] Kirschvink JL, Winklhofer M, Walker MM (2010) Biophysics of magnetic orientation: strengthening the interface between theory and experimental design. J R Soc Interface 7 [Suppl 2]:S179–S191. 20071390 10.1098/rsif.2009.0491.focus PMC2843999

[B41] Klimesch W (1999) EEG alpha and theta oscillations reflect cognitive and memory performance: a review and analysis. Brain Res Brain Res Rev 29:169–195. 10209231 10.1016/s0165-0173(98)00056-3

[B42] Klimesch W, Doppelmayr M, Russegger H, Pachinger T, Schwaiger J (1998) Induced alpha band power changes in the human EEG and attention. Neurosci Lett 244:73–76. 9572588 10.1016/s0304-3940(98)00122-0

[B43] Kobayashi A, Kirschvink JL (1995) Magnetoreception and EMF effects: sensory perception of the geomagnetic field in animals and humans In: Electromagnetic fields: biological interactions and mechanisms (Blank M, ed), pp 367–394. Washington, DC: American Chemical Society Books.

[B44] Kramer G (1953) Wird die Sonnenhohe bei der Heimfindeorientierung verwertet? J Ornithol 94:201–219. 10.1007/BF01922508

[B45] Landler L, Painter MS, Youmans PW, Hopkins WA, Phillips JB (2015) Spontaneous magnetic alignment by yearling snapping turtles: rapid association of radio frequency dependent pattern of magnetic input with novel surroundings. PLoS One 10:e0124728. 10.1371/journal.pone.0124728 25978736 PMC4433231

[B46] Levinson SC (2003) Space in language and cognition. Cambridge, UK: Cambridge University Press.

[B47] Light P, Salmon M, Lohmann KJ (1993) Geomagnetic orientation of loggerhead sea turtles: evidence for an inclination compass. J Exp Biol 182:1–10.

[B48] Liu GT (2005) The trigeminal nerve and its central connections In: Walsh & Hoyt’s clinical neuro-ophthalmalogy, Ed 6 (Miller NR, Newman NJ, Biousse V, Kerrison JB, eds), pp 1233–1268. Philadelphia, PA: Lippencott Williams & Wilkins.

[B49] Lohmann KJ, Lohmann CMF (1996) Detection of magnetic field intensity by sea turtles. Nature 380:59–61. 10.1038/380059a0

[B50] Lohmann KJ, Cain SD, Dodge SA, Lohmann CM (2001) Regional magnetic fields as navigational markers for sea turtles. Science 294:364–366. 10.1126/science.1064557 11598298

[B51] Maher BA, Ahmed IA, Karloukovski V, MacLaren DA, Foulds PG, Allsop D, Mann DM, Torres-Jardón R, Calderon-Garciduenas L (2016) Magnetite pollution nanoparticles in the human brain. Proc Natl Acad Sci USA 113:10797–10801. 10.1073/pnas.1605941113 27601646 PMC5047173

[B52] Martin H, Lindauer M (1977) Der Einfluss der Erdmagnetfelds und die Schwerorientierung der Honigbiene. J Comp Physiol 122:145–187. 10.1007/BF00611888

[B53] Meakins F (2011) Spaced out: intergenerational changes in the expression of spatial relations by Gurindji people. Aust J Linguist 31:43–77. 10.1080/07268602.2011.532857

[B54] Meakins F, Algy C (2016) Deadly reckoning: changes in Gurindji children’s knowledge of cardinals. Aust J Linguist 36:479–501. 10.1080/07268602.2016.1169973

[B55] Meakins F, Jones C, Algy C (2016) Bilingualism, language shift and the corresponding expansion of spatial cognitive systems. Lang Sci 54:1–13. 10.1016/j.langsci.2015.06.002

[B56] Merritt R, Purcell C, Stroink G (1983) Uniform magnetic-field produced by three, four, and five square coils. Rev Sci Instrum 54:879–882. 10.1063/1.1137480

[B57] Mora CV, Davison M, Wild JM, Walker MM (2004) Magnetoreception and its trigeminal mediation in the homing pigeon. Nature 432:508–511. 10.1038/nature03077 15565156

[B58] Munro U, Munro JA, Phillips JB, Wiltschko W (1997a) Effect of wavelength of light and pulse magnetisation on different magnetoreception systems in a migratory bird. Aust J Zool 45:189–198. 10.1071/ZO96066

[B59] Munro U, Munro JA, Phillips JB, Wiltschko R, Wiltschko W (1997b) Evidence for a magnetite-based navigational “map” in birds. Naturwissenschaften 84:26–28. 10.1007/s001140050343

[B60] Nuwer MR, Comi G, Emerson R, Fuglsang-Frederiksen A, Guérit JM, Hinrichs H, Ikeda A, Luccas FJ, Rappelsburger P (1998) IFCN standards for digital recording of clinical EEG. International Federation of Clinical Neurophysiology. Electroencephalogr Clin Neurophysiol 106:259–261. 9743285 10.1016/s0013-4694(97)00106-5

[B61] Peng W, Hu L, Zhang Z, Hu Y (2012) Causality in the association between P300 and alpha event-related desynchronization. PLoS One 7:e34163. 10.1371/journal.pone.0034163 22511933 PMC3325251

[B62] Pfurtscheller G, Lopes da Silva FH (1999) Event-related EEG/MEG synchronization and desynchronization: basic principles. Clin Neurophysiol 110:1842–1857. 10.1016/S1388-2457(99)00141-8 10576479

[B63] Pfurtscheller G, Neuper C, Mohl W (1994) Event-related desynchronization (ERD) during visual processing. Int J Psychophysiol 16:147–153. 8089033 10.1016/0167-8760(89)90041-x

[B64] Poisson RJ, Miller ME (2014) Spatial disorientation mishap trends in the U.S. Air force 1993-2013. Aviat Space Environ Med 85:919–924. 10.3357/ASEM.3971.2014 25197890

[B65] Ritz T, Adem S, Schulten K (2000) A model for photoreceptor-based magnetoreception in birds. Biophys J 78:707–718. 10.1016/S0006-3495(00)76629-X 10653784 PMC1300674

[B66] Rosenblum B, Jungerman RL, Longfellow L (1985) Limits to induction-based magnetoreception In: Magnetite biomineralization and magnetoreception in organisms: a new biomagnetism (Kirschvink JL, Jones DS, MacFadden BJ, eds), pp 223–232. New York, NY: Plenum Press.

[B67] Saper CB (2002) The central autonomic nervous system: conscious visceral perception and autonomic pattern generation. Annu Rev Neurosci 25:433–469. 10.1146/annurev.neuro.25.032502.111311 12052916

[B68] Sastre A, Graham C, Cook MR, Gerkovich MM, Gailey P (2002) Human EEG responses to controlled alterations of the Earth’s magnetic field. Clin Neurophysiol 113:1382–1390. 10.1016/S1388-2457(02)00186-4 12169319

[B69] Schulten K (1982) Magnetic-field effects in chemistry and biology. Festkorperprobleme 22:61–83.

[B70] Schultheiss-Grassi PP, Wessiken R, Dobson J (1999) TEM investigations of biogenic magnetite extracted from the human hippocampus. Biochim Biophys Acta 1426:212–216. 9878742 10.1016/s0304-4165(98)00160-3

[B71] Schwarze S, Schneider NL, Reichl T, Dreyer D, Lefeldt N, Engels S, Baker N, Hore PJ, Mouritsen H (2016) Weak broadband electromagnetic fields are more disruptive to magnetic compass orientation in a night-migratory songbird (*Erithacus rubecula*) than strong narrow-band fields. Front Behav Neurosci 10:55. 27047356 10.3389/fnbeh.2016.00055 PMC4801848

[B72] Semm P, Beason RC (1990) Responses to small magnetic variations by the trigeminal system of the bobolink. Brain Res Bull 25:735–740. 2289162 10.1016/0361-9230(90)90051-z

[B73] Tomanova K, Vacha M (2016) The magnetic orientation of the Antarctic amphipod *Gondogeneia antarctica* is cancelled by very weak radiofrequency fields. J Exp Biol 219:1717–1724. 10.1242/jeb.132878 27026715

[B74] Tukey JW (1949) Comparing individual means in the analysis of variance. Biometrics 5:99–114. 18151955

[B75] Veniero D, Bortoletto M, Miniussi C (2009) TMS-EEG co-registration: on TMS-induced artifact. Clin Neurophysiol 120:1392–1399. 10.1016/j.clinph.2009.04.023 19535291

[B76] Walker MM, Diebel CE, Haugh CV, Pankhurst PM, Montgomery JC, Green CR (1997) Structure and function of the vertebrate magnetic sense. Nature 390:371–376. 20358649 10.1038/37057

[B77] Walker MM, Dennis TE, Kirschvink JL (2002) The magnetic sense and its use in long-distance navigation by animals. Curr Opin Neurobiol 12:735–744. 12490267 10.1016/s0959-4388(02)00389-6

[B78] Wegner RE, Begall S, Burda H (2006) Magnetic compass in the cornea: local anaesthesia impairs orientation in a mammal. J Exp Biol 209:4747–4750. 10.1242/jeb.02573 17114407

[B79] Welch PD (1967) The use of fast Fourier transform for estimation of power spectra: a method based on time averaging over short modified periodograms. IEEE Trans Audio Electroacoust 15:70–73. 10.1109/TAU.1967.1161901

[B80] Westby GWM, Partridge KJ (1986) Human homing: still no evidence despite geomagnetic controls. J Exp Biol 120:325–331. 3958671 10.1242/jeb.120.1.325

[B81] Wiltschko R, Wiltschko W (1995a) Magnetic orientation in animals. Berlin: Springer.

[B84] Wiltschko W, Wiltschko R (1995b) Migratory orientation of European robins is affected by the wavelength of light as well as by a magnetic pulse. J Comp Physiol A 177:363–369.

[B82] Wiltschko R, Thalau P, Gehring D, Niessner C, Ritz T, Wiltschko W (2015) Magnetoreception in birds: the effect of radio-frequency fields. J R Soc Interface 12:20141103. 10.1098/rsif.2014.1103 PMC4305412 25540238

[B83] Wiltschko W (1972) The influence of magnetic total intensity and inclination on directions preferred by migrating European robins (*Erithacus rubeccula*). In: Animal orientation and navigation (Galler SR, Schmidt-Koenig K, Jacobs GJ, Belleville RE, eds), pp 569–578. Washington, DC: U.S. Government Printing Office.

[B85] Wiltschko W, Munro U, Beason RC, Ford H, Wiltschko R (1994) A magnetic pulse leads to a temporary deflection in the orientation of migratory birds. Experientia 50:697–700. 10.1007/BF01952877

[B86] Wiltschko W, Munro U, Ford H, Wiltschko R (1998) Effect of a magnetic pulse on the orientation of silvereyes, zosterops l. lateralis, during spring migration. J Exp Biol 201:3257–3261. 9808838 10.1242/jeb.201.23.3257

[B87] Wiltschko W, Munro U, Wiltschko R, Kirschvink JL (2002) Magnetite-based magnetoreception in birds: the effect of a biasing field and a pulse on migratory behavior. J Exp Biol 205:3031–3037. 12200406 10.1242/jeb.205.19.3031

[B88] Wiltschko W, Ford H, Munro U, Winklhofer M, Wiltschko R (2007) Magnetite-based magnetoreception: the effect of repeated pulsing on the orientation of migratory birds. J Comp Physiol A Neuroethol Sens Neural Behav Physiol 193:515–522. 10.1007/s00359-006-0207-5 17318656

[B89] Wiltschko W, Munro U, Ford H, Wiltschko R (2009) Avian orientation: the pulse effect is mediated by the magnetite receptors in the upper beak. Proc Biol Sci 276:2227–2232. 10.1098/rspb.2009.0050 19324756 PMC2677601

[B90] Winklhofer M, Kirschvink JL (2010) A quantitative assessment of torque-transducer models for magnetoreception. J R Soc Interface 7 [Suppl 2]:S273–S289. 10.1098/rsif.2009.0435.focus 20086054 PMC2843997

[B91] Yeagley HL (1947) A preliminary study of a physical basis of bird navigation. J Appl Phys 18:1035–1063. 10.1063/1.1697587

